# Shoot tip necrosis of in vitro plant cultures: a reappraisal of possible causes and solutions

**DOI:** 10.1007/s00425-020-03449-4

**Published:** 2020-09-03

**Authors:** Jaime A. Teixeira da Silva, Esmaeil Nezami-Alanagh, María E. Barreal, Mafatlal M. Kher, Adhityo Wicaksono, Andrea Gulyás, Norbert Hidvégi, Katalin Magyar-Tábori, Nóra Mendler-Drienyovszki, László Márton, Mariana Landín, Pedro Pablo Gallego, John A. Driver, Judit Dobránszki

**Affiliations:** 1 Present Address: Miki-cho Post Office, 3011-2, P. O. Box 7, Ikenobe, Kagawa-ken 761-0799 Japan; 2 Research Institute of Nyíregyháza, IAREF, University of Debrecen, P. O. Box 12, Nyíregyháza, 4400 Hungary; 3 Department of Plant Biology and Soil Science, Faculty of Biology, University of Vigo, 36310 Vigo, Spain; 4 Pinar Biotech. Co., Ltd., East Azarbaijan Science and Technology Park , Tabriz, Iran; 5 School of Science (SOS), GSFC University, P. O. Fertilizernagar, Vadodara, 391750 Gujarat India; 6 Division of Biotechnology, Generasi Biologi Indonesia (Genbinesia) Foundation, Jl. Swadaya Barat No. 4, Gresik Regency, 61171 Indonesia; 7 Department of Pharmacology, Pharmacy and Pharmaceutical Technology, Faculty of Pharmacy, University of Santiago, Santiago de Compostela, Spain; 8 Driver Consulting Inc., 2601 Tim Bell Road, Waterford, CA 95386 USA

**Keywords:** Boron, Calcium, Chloride, In vitro shoots, Mineral nutrient deficiency, Physiological disorder, Plant growth regulators

## Abstract

**Main conclusion:**

Shoot tip necrosis is a physiological condition that negatively impacts the growth and development of in vitro plant shoot cultures across a wide range of species.

**Abstract:**

Shoot tip necrosis is a physiological condition and disorder that can arise in plantlets or shoots in vitro that results in death of the shoot tip. This condition, which can spread basipetally and affect the emergence of axillary shoots from buds lower down the stem, is due to the cessation of apical dominance. STN can occur at both shoot multiplication and rooting stages. One of the most common factors that cause STN is nutrient deficiency or imbalance. Moreover, the presence or absence of plant growth regulators (auxins or cytokinins) at specific developmental stages may impact STN. The cytokinin to auxin ratio within an in vitro plant can be modified by varying the concentration of cytokinins used in the culture medium. The supply of nutrients to in vitro shoots or plantlets might also affect their hormonal balance, thus modifying the occurrence of STN. High relative humidity within culture vessels and hyperhydricity are associated with STN. An adequate supply of calcium as the divalent cation (Ca^2+^) can hinder STN by inhibiting the accumulation of phenolic compounds and thus programmed cell death. Moreover, the level of Ca^2+^ affects auxin transport and ethylene production, and higher ethylene production, which can occur as a result of high relative humidity in or poor ventilation of the in vitro culture vessel, induces STN. High relative humidity can decrease the mobility of Ca^2+^ within a plant, resulting in Ca^2+^ deficiency and STN. STN of in vitro shoots or plantlets can be halted or reversed by altering the basal medium, mainly the concentration of Ca^2+^, adjusting the levels of auxins or cytokinins, or modifying culture conditions. This review examines the literature related to STN, seeks to discover the associated factors and relations between them, proposes practical solutions, and attempts to better understand the mechanism(s) underlying this condition in vitro.

**Electronic supplementary material:**

The online version of this article (10.1007/s00425-020-03449-4) contains supplementary material, which is available to authorized users.

## Introduction

**Fig. 1:**
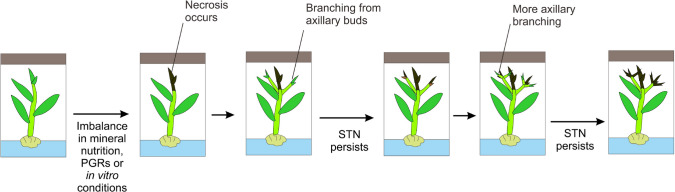
Schematic diagram of shoot tip necrosis (STN). An imbalance in minerals, nutrients, plant growth regulators, or other in vitro conditions, lead to STN. This results in the blackening and death of the terminal shoot tip, the branching of axillary buds, and in some cases, STN in axillary shoots, leading to the formation of a “witches’ broom” pattern

Shoot tip necrosis (STN) is a term that was originally coined by Sha et al. (1985). STN is sometimes also referred to as shoot tip abortion (Millington 1963), tip burn (McCown and Sellmer 1987), apical necrosis (Amo-Marco and Lledo 1996; Koubouris and Vasilakakis 2006; Machado et al. 2014), apex necrosis (Rugini et al. 1986), top necrosis (De Klerk and ter Brugge 2011), shoot tip damage/injury (Ahmed and Palta 2017b), or shoot die-back (Barghchi and Alderson 1996). STN occurs when the shoot tip of a plant, both ex vitro and in vitro, shows signs of browning and death during multiplication, elongation and/or rooting stages, despite growing in apparently ideal conditions (Vieitez et al. 1989; Bairu et al. 2009b). In vitro, STN can ultimately result in the inhibited growth of the entire plantlet or it can be localized at affected shoots. The affected area spreads basipetally down from the shoot tip to lower parts of shoots. However, shoot formation from basal axillary shoot buds is not necessarily inhibited, as was observed for pistachio (*Pistachia vera* L.) (Barghchi and Alderson 1996). STN is also not always fatal to the plant, and apical dominance can be assumed by the next closest axillary bud, at least in sweet chestnut (*Castanea sativa* Mill.) and oak (*Quercus robur* L.) (Vieitez et al. 1989). If growing axillary branches develop STN, then a “witches’ broom” pattern develops (Fig. 1; McCown and Sellmer 1987). On some occasions, the shoot tip can outgrow STN, leaving behind a scarred part of the stem with deformed leaves (McCown and Sellmer 1987). Sudha et al. (1998) observed axillary branching after STN in jivanthi (*Holostemma annulare* (Robx.) K. Schum., i.e., *Holostemma ada-kodien* Schult.) in vitro cultures. STN is problematic not only for stock cultures of in vitro plantlets, but also for commercial production (Sha et al. 1985).

The precise mechanism underlying STN still remains unclear, although some possible reasons have been proposed, including mineral deficiency or the presence of high concentrations of plant growth regulators (PGRs) in the medium. One of the most cited reasons is calcium (Ca) deficiency. Ca deficiency is also a reason for the tip-burn disorder in the leaves and stems of field-grown fruits such as strawberry (*Fragaria* × *ananassa* Duchesne) and vegetables (Mason and Guttridge 1974, 1975; Saure 1998) and its symptoms closely resemble those of STN. This review aims to examine the literature that exists on this physiological disorder, including an earlier review by Bairu et al. (2009b), while exploring new literature published over the past decade. One objective is to attempt to better identify some of the possible reasons for the occurrence of STN and to suggest practical solutions to alleviate this physiological disorder in vitro.

Shoot tips are a popular explant in plant tissue culture. On occasion, shoot tip explants necrose (e.g., Krishna et al. 2008). In this review, the necrosis of shoot tip explants, i.e., explant necrosis, is not considered to be STN, which relates exclusively to the shoot tip of a tissue-cultured in vitro plantlet.

## Shoot tip necrosis: occurrence and alleviation

**Fig. 2:**
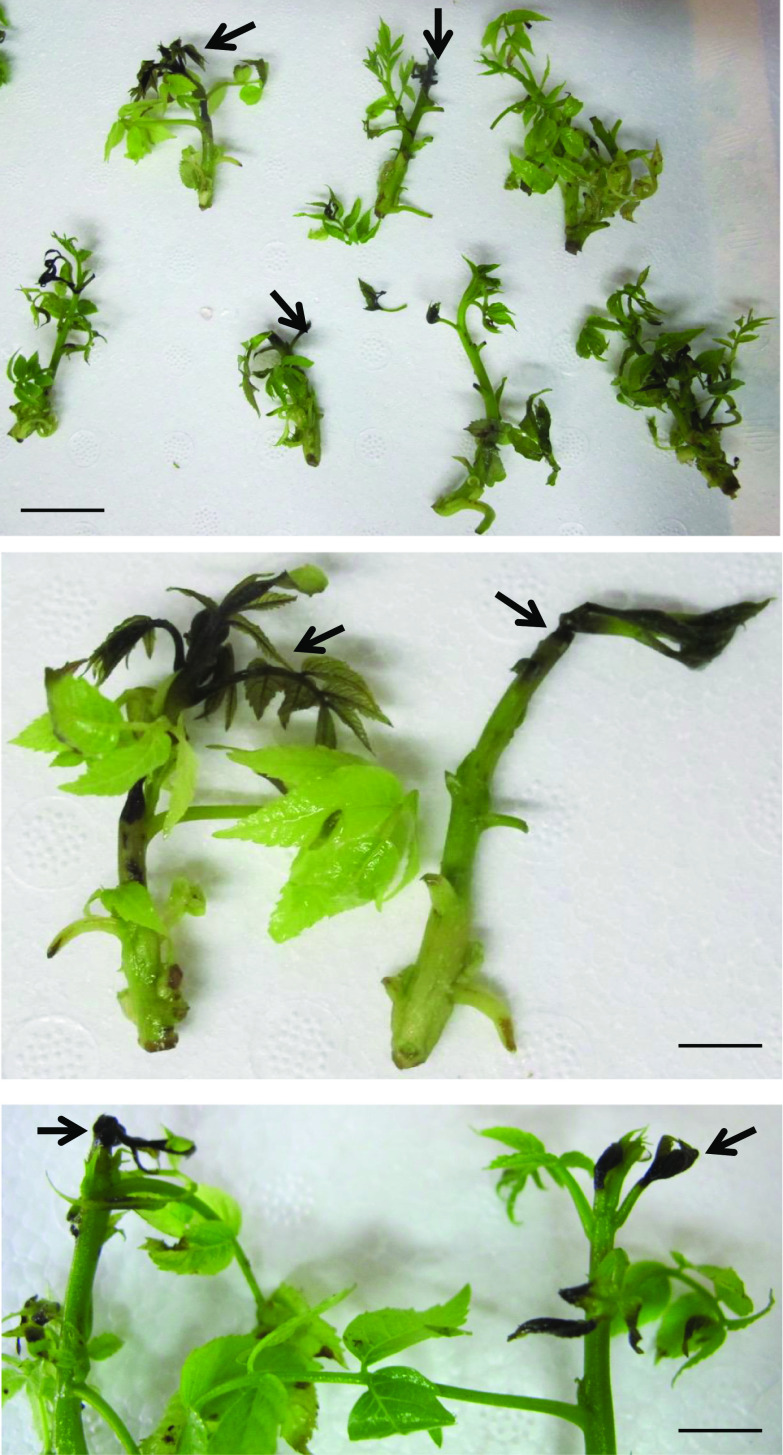
Incidence of shoot tip necrosis (STN) in in vitro cultures of walnut (*Juglans regia* L.) Paradox rootstock during micropropagation in Driver and Kuniyuki walnut medium (DKW; Driver and Kuniyuki, 1984) (unpublished results). (1) if 3-week-old shoots were used, the incidence of STN was high (20–30%), most likely because tissue is soft (non-lignified), but the use of 4-week-old shoots, which are more lignified, have a lower incidence of STN, even reduced to 0%; (2) initial “Vlach” [a selection of Paradox (*J. hindsii* x *J. regia*)] material is from a 110-year-old mother tree, located near Modesto (CA, USA) for which in vitro cultures were originally established by John Driver in 1985; (3) walnut tends to be somewhat recalcitrant to rooting, so occasionally high concentrations of IBA (8–10 mg/l) are added to rooting medium. If IBA is transported to the shoot tip, especially soft shoots that may take up excessive amounts of IBA, this may result in the death to the shoot tip, a condition we coin as “IBA burn”, which is visually similar to STN. However, this does not take place if more mature shoots are used and this can be achieved by increasing the subculture interval from 3 to 4 weeks. Black arrows indicate STN. Scale bars indicate 3 cm (top), 2 cm (middle) and 2 cm (bottom)

A wide range of plants display STN in in vitro cultures (Table 1; Suppl. Table 1). Among all published studies, the occurrence of STN is particularly prominent in trees and woody shrubs (58.9% and 21.9%, respectively, of studies in Suppl. Table 1; Suppl. Figure 1). Studies on pistachio represent the largest proportion (10.8%) of studies on STN in vitro, followed by pear (*Pyrus* spp.) (8.1%) (Suppl. Figure 2). The incidence of STN in micropropagation, especially at the rooting stage, is shown in Fig. 2. STN, at least according to the reported literature, has occurred most frequently in the Rosaceae (20.5%), followed by the Anacardiaceae (12.3%) (Suppl. Figure 3). We caution readers that relative values might simply indicate the popularity of a studied species and not necessarily the actual incidence of STN in plan species or families studied to date. For example, only a single report on STN exists for an orchid, hybrid *Cymbidium* (Guha and Usha Rao 2012), so the incidence for the Orchidaceae is in fact 100% of studies, but the relative incidence (relative to all other species studied in Table 1) is only 1.4%.

**Table 1:** Shoot tip necrosis: observations and possible solutions*

Scientific name and cultivar	Stage, medium and observed problems	Reason(s) provided for incidence of STN	Solution provided to halt, reduce, or prevent STN, and other observations	References
*Azadirachta indica* A. Juss	Optimum SMM: MS + 1.11 µM BA + 1.43 µM IAA + 81.43 µM AdS. STN = UQ	Basal medium micro- and macronutrient concentration	Addition of 0.42 mM Ca(NO_3_)_2_, 0.70 mM Na_2_SO_4_, and 0.57 mM K_2_SO_4_	Arora et al. (2010)
*Begonia homonyma* Steud	Optimum SMM: MS + 15 µM BA + 5 µM NAA. STN (all on MS) = 18% (15 µM *m*TR + 5 µM NAA for 12 weeks → 2 µM *m*TR + 0.5 µM NAA for 6 weeks), 34% (15 µM TDZ + 5 µM NAA for 12 weeks → 2 µM TDZ + 0.5 µM NAA for 6 weeks), 44% (15 µM BA + 5 µM NAA for 12 weeks → 2 µM BA + 0.5 µM NAA for 6 week)	Use of PGRs. High BA conc. and/or use of BA as the CK	Use of *m*TR. Use half-strength MS rather than MS, reduce BA conc. to 0.5 µM and add 2 or 5 µM GA_3_: STN = 10–36% in various media defined in column 2	Kumari et al. (2017)
*Butea monosperma* (Lam.) Taub	Optimum SGM: half-strength WPM + 5 mg/l BA (± 10 mg/l fructose). 90% STN in terminal 2–3 mm	No substantiated reason provided. Only theoretical observation with no supporting data	Addition of fructose eliminated STN in 95% of STN-positive cultures. Some phenolics were exuded from cut ends	Kulkarni and D’Souza (2000)
*Castanea dentata* (Marsh.) Borkh. cv. B’ville, Iowa #2, BDW	Optimum RIM: half-strength MS + AC. SEM: WPM salts + NN vitamins. SMM = 500 mg/l PVP 40 + 500 mg/l MES + 0.89 µM BA. STN = 25–67%, depending on genotype and treatment. STN reduced to 19–21% across three genotypes in replication trial	Wounding, developmental stage, genotype	Low concentration of BA (0.22 µM) at an advanced stage of root initiation reduced STN. When on SEM, wounding had no effect on STN (~ 30–38%, 13–30%, 20–25% for B’ville, Iowa #2, and BDW, respectively). STN increased to 67% and 88% in Iowa #2 and BDW, respectively, when cuttings were plated on SMM (no change for B’ville, at 38%)	Xing et al. (1997)
*Castanea sativa* Mill. clones 431, T-13, 812; *Quercus robur* L	Optimum RIM: half-strength MS + 3 mg/l IBA (7 day) or dip in 1 g/l IBA (20–60 s) for chestnut; half-strength Gresshoff and Doy (1972) basal + 0.5 g/l IBA (8 min) for oak. STN UQ (only axillary shoot development)	In SEM, when BA was removed, or in RIM, STN developed	Addition of 0.01 mg/l BA to RIM, but this reduced rooting in chestnut and oak, but axillary shoots developed marginally more (+ 1%) in chestnut clone T-13. When the cut surface of shoot tips was added to BA-impregnated agar, rooting was reduced in both trees, but axillary shoot development increased, the amount depending on the day of decapitation	Vieitez et al. (1989)
*Castanea sativa* Mill. cv. Garrone rosso, Clone 46	Optimum RIM: MS + 0.044 µM BA + 5 µM IBA (8 day) then same medium without IBA. STN = 23% after 8 day, 77% after 26 day for Clone 46; UQ for Garrone rosso	Ca deficiency; lack of BA	Clone 46 formed > twofold more STN than Garrone rosso (68% vs 25%). A block of agar containing 3 mM CaCl_2_ and/or 5 µM BA that was placed around shoot tips eliminated or delayed STN	Piagnani et al. (1996)
*Cercis canadensis* var. *mexicana*	SMM: WPM + MS vitamins + 11.1 µM BA. STN UQ	Excessively high concentrations of 2iP (25–74 µM), TDZ (presumably 5 or 11–23 µM) and kinetin (concentration range NR)	General suggestions on how to improve shoot growth, but no specific details, or data, about how to improve STN	Mackay et al. (1995)
*Corylus avellana *L	Optimum SMM: half-strength Cheng (1975) basal + 25 µM BA (15 days) → same medium but + 0.5 or 2.5 µM BA (25 days); optimum RIM: half-strength liquid Cheng (1975) basal + 50 µM IBA (5 days) → same medium (solid) (15 days). STN = UQ	High IBA concentration	Reducing IBA from 50 µM at the rooting stage to 10 or 25 µM, or by reducing exposure period to IBA to 8 days	Pérez et al. (1985)
*Cydonia oblonga* Mill. rootstock clone C	Optimum SMM: MS + 5 µM BA. STN = 15% in SM of control cultures	Ca deficiency	Raising Ca^2+^ (in the form of Ca(NO_3_)_2_) from 3 to 18 mM reduced STN, but this also reduced shoot proliferation. Link between Ca deficiency and hyperhydricity unclear	Singha et al. (1990)
*Cymbidium* hybrid Via del Playa Yvonne	Optimum SMM: MS – MgSO_4_ + Na_2_SO_4_. STN = 5% (control), 35%, 60%, 80% (10, 15, 20 µM SNP, respectively)	Addition of SNP, a nitric oxide donor	Nitric oxide, a positive and negative regulator of stress, could not prevent STN	Guha and Usha Rao (2012)
*Dalbergia latifolia* Roxb	Optimum SMM: ¾ (macro) MS or WPM + 5 mg/l BA + 0.5 mg/l NAA. STN = UQ	Tended to find STN associated with leaf abscission, but not linked to poor aeration or high humidity	Doubling Ca^2+^ concentration in MS or WPM media did not reduce STN. Solution only provided to reduce leaf abscission by adjusting the NH_4_/NO_3_ ratio, but not STN	Lakshmi Sita and Raghava Swamy (1993)
*Dipterocarpus alatus* Roxb., *D. intricatus* Dyer	Optimum seedling establishment: MS or WPM + 0.1 µM BA. STN = UQ	Nitrogen level	Removal of NH_4_NO_3_ from WPM. High humidity likely not the cause because of high aeration of vessels	Linington (1991)
Dwarf rose (*Rosa gymnocarpa* Nutt.) cv. Starina	Optimum RIM: auxin-free MS. Lowest incidence of STN = 6% (on RIM). When 1 mg/l IAA was added, STN increased from 6–22% to 16–62% (range caused by the treatment)	Inclusion of auxin, specifically IAA	In the absence of auxin, 2.5–10 mg/l AgNO_3_ reduced the incidence of STN from 22% to 2–12%. In IAA-containing RIM, 1.5 × Ca^2+^ levels decreased STN from 58 to 28%. In IAA-containing RIM, 1.5 × Ca^2+^ levels + 2 × Mg^2+^ levels decreased STN from 58 to 24%	Podwyszyńska and Goszczyńska (1998)
*Ensete ventricosum* Welw. cv. Oniya	Optimum SMM: MS + 11 µM BA + 6 µM IAA	The term STN was not used	However, STN was induced since shoot tips were split vertically down the center for micropropagation. 40% of greenhouse-derived shoot tips died due to blackening (*aka* STN; 0% in in vitro shoot tips)	Diro and van Staden (2005)^a^
*Gaultheria hispidula* (L.) Muhl. ex Bigelow, *Rhododendron* cv. *Chinsayii*, *Rhododendron dauricum* L	Optimum SMM: Anderson (1984) basal + 15.9–16.3 mg/l 2iP (across three plants). STN = UQ	Presence of BA in medium at any concentration (0.1–10 mg/l). Use of 2iP did not induce STN	No suggestions	Norton and Norton (1985)
*Haloxylon persicum* (Bunge ex Boiss & Buhse)	Optimum SMM: MS + 0.5 µM TDZ. STN = 100% (0 or 2 µM TDZ), 86% (0.5 µM TDZ), 90% (1 µM TDZ); all with 10 µM Kin: 74% (2 mM CaCl_2_), 65% (4 mM CaCl_2_), 21% (2 mM CaCl_2_ + 0.1 mM H_3_BO_3_), 16% (4 mM CaCl_2_ + 0.1 mM H_3_BO_3_), 23% (2 mM CaCl_2_ + 0.2 mM H_3_BO_3_), 19% (4 mM CaCl_2_ + 0.2 mM H_3_BO_3_)	Low Ca^2+^ and ${\text{BO}}_{3}^{ - }$	Addition of 4 mM CaCl_2_ + 0.1 mM H_3_BO_3_ + 10 µM Kin. Use of several sugars (sucrose and maltose (60–180 mM), or fructose and glucose (110–330 mM)) with 10 µM Kin did not reduce STN (range = 89–100% for all treatments), except for 120 mM sucrose (STN reduced to 84%)	Kurup et al. (2018)
*Harpagophytum procumbens* [(Burch) de Candolle ex Meissner]	Optimum SMM: half-strength MS + 6 mM Ca^2+^. STN = 27% (PGR-free MS), 25–35% (MS + 5 µM BA, *m*T or *m*TR), 33–62% (MS + 5 or 10 µM BA, *m*T or *m*TR + 2.5 µM IAA), 80% (half-strength MS), 20–133% (half-strength MS + 6–9 mM Ca^2+^ alone or in various combinations with 0.2–0.5 mM ${\text{BO}}_{3}^{ - }$)	High CK (BA) level. Addition of auxin (IAA)	Addition of 6–9 mM Ca^2+^ with or without 0.2–0.5 mM ${\text{BO}}_{3}^{ - }$, or only 0.5 mM ${\text{BO}}_{3}^{ - }$, and in IAA-containing medium, 5 or 10 µM *m*T or *m*TR reduced STN	Bairu et al. (2009a)
*Harpagophytum procumbens* [(Burch) de Candolle ex Meissner]	Optimum SMM: MS + 8.8 µM BA. STN = 88, 90, 86% (full-strength MS, NN and WPM); 29, 27, 27% (half-strength MS, NN and WPM); 14, 26, 28% (quarter-strength MS, NN and WPM); 18, 21, 25, 26% (1, 2, 3, 4% sucrose); 29, 37, 64, 76% (sucrose, glucose, fructose, maltose at 0.086 M); 19, 28% (2-week subculture; 4-week continuous culture)	High mineral content of MS, NN, or WPM. High sucrose concentration (> 3%). Use of non-sucrose sources or carbohydrates. Lack of subculture	Reducing basal media to half strength. Use of low sucrose concentration. Use of 2-week subcultures	Jain et al. (2009)
*Harpagophytum procumbens* [(Burch) de Candolle ex Meissner]	Same as Bairu et al. (2009a)	Active CKs may be converted to other inactive or irreversible forms of CKs, e.g., 9-glucosides	Selection of CK, and the choice of CK:auxin ration can influence endogenous level of CKs, and thus the outcome of STN	Bairu et al. (2011)
*Harpagophytum procumbens* [(Burch) de Candolle ex Meissner]	Optimum SMM: MS + 1.5 mg/l BA + 6.2 mg/l H_3_BO_3_. STN = 53% (SMM + 10 mg/l H_3_BO_3_), 13% (SMM + 10 mg/l H_3_BO_3_ + 5 mM Si in the form of sodium silicate solution)	No reason provided	Addition of Si as SiO_2_	Lišková et al. (2016)
*Hibiscus rosa-sinensis* L. cv. Cassiopeia Wind Yellow, Caribbean Pink	Optimum SMM: MS + 2.2 µM BA. STN = UQ	Low Ca^2+^ level (independent of BA concentration)	STN only assessed visually, but photographic evidence provided	Christensen et al. (2008)
*Juglans nigra* L	Optimum SMM: half-strength DKW. STN = 11% or 17% in stage 1 (3–4 week culture) when Zea = 5 or 12.5 µM, respectively, (44% and 33% in stage 2, which is 5–8 week of culture). 53% STN on MS when 12.5 µM Zea was used, and measured in stage 2	Use of BA at 25 µM or Zea at 12.5 or 25 µM. Basal medium (decreasing level of STN): half-strength DKW > DKW > MS > WPM (stage 1) or MS > half-strength DKW > DKW > WPM (stage 2)	Increasing TDZ from 0.5 to 1 µM or reducing BA from 25 to 12.5 µM BA improved percentage of spontaneous shoots, i.e., reduced STN	Bosela and Michler (2008)
*Lavandula angustifolia* Mill. cv Provence Blue	Optimum SMM: MS + 1 µM BA (40 day subculture). STN = 10% (1320 mg/l CaCl_2_), 21% (440 mg/l CaCl_2_) for a single subculture; 30% (1320 mg/l CaCl_2_), 51% (440 mg/l CaCl_2_) for a second subculture	Low Ca^2+^ level. Subcultures	Including CaCl_2_ at 1320 mg/l. Only subculture once	Machado et al. (2014)
*Lens culinaris* Medikus cv. Titore	Optimum SMM: MS + 0.4–0.8 mg/l. STN = 85%, 70%, 56% and 49% in MS + 0.2, 0.4, 0.6 and 0.8 mg/l BA, respectively (91%, 87%, 75% and 73% in B5; 0–3% in MS + 440 mg/l CaCl_2_; 18%, 16%, 5% and 5% in B5 + 750 mg/l CaCl_2_)	Low BA conc. or reduced levels of Ca^2+^	Increasing BA conc. or adding 750 mg/l CaCl_2_ to basal medium	Ye et al. (2002)
*Lonicera caerulea* f. *caerulea*; *L. caerulea* f. *edulis*	SMM: 9/10 × MS + 8.9 µM BA, 2.4 µM pyridoxine HCl. STN = 17% on half-strength MS; 6% on 75% MS; 9% on MS	Insufficient micro- and macronutrients in MS; high day/night temperatures	In *caerulea* form, 0% STN at 24 °C/20 °C (6% at 26 °C/20 °C, 17% at 28 °C/21 °C). In *edulis* form, 1% STN at 24 °C/20 °C (23% at 26 °C/20 °C, 49% at 28 °C/21 °C)	Karhu (1997)
*Macadamia tetraphylla* L.A.S.Johnson	Optimum SMM: MS + 2 mg/l BA. RIM: SM + 3 mg/l IBA. STN in RIM: 40% at 3 mM Ca^2+^ (20%, 70%, 85% at 6, 12 and 24 mM). Mulwa and Bhalla (2000) reported 76% STN in RIM	Inadequate aeration, high humidity	Application of < 6 mM Ca^2+^ in RIM	Mulwa and Bhalla (2000); Bhalla and Mulwa (2003)
*Malus* × *domestica* (Borkh.); *Camellia sinensis* (L.) Kuntze; *Populus tremula* L. × *P. alba* L.; *Gerbera jamesonii* Bolus ex Hooker f	SMM/RIM: MS + 2.2 µM BA + 5.3 µM NAA. STN = 49, 53, 3 and 5% in shoots of apple, tea, poplar and gerbera, respectively	Lack of exogenous CK (BA) in medium; lack of endogenous hormones in plants	CK required in medium but details not provided	Kataeva et al. (1991)
*Musa* spp. cv. Grande Naine (GN; AAA), Dwarf Cavendish (DC; AAA), Nendran (AAB), Quintal Nendran (QN; AAB)	Optimum SMM: MS + 6.66 µM BA. STN = 27% and 29% in GN and DC after seven subcultures on SMM (18% and 19% at the rooting stage); 38% and 40% in Nendran and QN after five subcultures on SMM (26% and 27% at the rooting stage)	Low Ca^2+^ level	Reducing the culture period, modifying salt strength in basal medium, addition of various PGRs (Kin, NAA, and IBA), adjusting levels of sucrose, fructose, and AgNO_3_ did not improve STN levels. Addition of 50–100 mg/l CaCl_2_ for at least two subcultures after the fourth and sixth subculture (for bananas and plantains, respectively) allowed 91–97% of shoots (across all four cultivars) to be recovered (unclear if recovered shoots were free of STN)	Martin et al. (2007)
*Paeonia suffruticosa* Andr	SMM: WPM + 0.3% AC. STN UQ	Low Ca^2+^ level	Adding 6 mM CaCl_2_ to WPM	Wang and van Staden (2001)
*Pimelea spicata* R.Br	Optimum SMM: half-strength MS + 0.5 or 1.0 mg/l BA. STN = 38% (MS), 73% (MS + ventilation), 18% (half-strength MS), 56% (half-strength MS + ventilation), 32% (half-strength MS + 440 mg/l CaCl_2_)	Addition of Ca^2+^. Application of ventilation to culture flasks	Using half-strength MS; not ventilating flasks; not adding supplementary Ca^2+^	Offord and Tyler (2009)
*Pistacia integrima* × *P. atlantica* rootstock UCB1	Optimum SMM: MS + 0.5 mg/l BA. STN = 42% (1 × CaCl_2_, 1 × H_3_BO_3_), 29% (1 × CaCl_2_, 2 × H_3_BO_3_), 38% (1 × CaCl_2_, 3 × H_3_BO_3_), 21% (1.5 × CaCl_2_), 19% (1.5 × CaCl_2_, 2 × H_3_BO_3_), 32% (1.5 × CaCl_2_, 3 × H_3_BO_3_), 19% (2 × CaCl_2_), 17% (2 × CaCl_2_, 2 × H_3_BO_3_), 30% (2 × CaCl_2_, 3 × H_3_BO_3_) (all × levels relative to MS)	Low Ca^2+^and ${\text{BO}}_{3}^{ - }$	Increasing CaCl_2_ level to 3 × MS level, and doubling MS level of H_3_BO_3_ reduced STN to 17%. High level of KNO_3_ (2280 mg/l) with 1320 or 1650 mg/l NH_4_NO_3_ eliminated STN from 10% at all other concentrations	Nezami et al. (2015)
*Pistacia integrima* × *P. atlantica* rootstock UCB1	Optimum SMM: MS + 0.5 mg/l BA, Gamborg vitamins. STN = 41% (control, no CNTs), 37% (50 µg/l CNTs), 30% (100 µg/l CNTs), 23% (150 µg/l CNTs), 13% (200 µg/l CNTs)	CNTs promote or improve physiological processes	Use of 200 µg/l CNTs	Kermani et al. (2017)
*Pistacia vera* L. cv. Mateur	SMM: MS + 3 mg/l BA tested for STN after 5 week. STN = 100% in 28-day cultures	Ca Ca^2+^ and ${\text{BO}}_{3}^{ - }$ deficiency	First STN symptoms in 12 days, affecting the whole aerial portion by 16 days. 100, 500 or 1000 µM of B, as H_3_BO_3_, reduced STN but 200 µM increased STN. 500 and 1000 µM B stunted shoots. Ca^2+^ as 0.3 and 3 mM CaCl_2_, and 15 and 30 mM CG increased shoot number and length, but only 15 and 30 mM could reduce STN, but eliminate it. Shoots immersed in liquid medium + 15 mM Ca^2+^ prevented STN	Abousalim and Mantell (1994)
*Pistacia vera* L. cv. NR	SMM: unrooted shoots on MS + 4 mg/l BA after 4 week. STN = partly quantified	High humidity in culture jars slowing nutrient flow	Addition of 12 mM CaCl_2_ reduced STN the most from 2.7/cultured explant to 1.1/cultured explant, but 3–24 mM was an effective range. Ca acetate could also reduce STN but also caused shoot stunting. H_3_BO_3_ at 100–800 µM reduced STN from 2.6 (100 µM) to 0.4 (800 µM), but above 200 µM, shoot multiplication was reduced while shoot stunting occurred at 100 and 200 µM. No Ca- or B-free controls were used. Increasing ventilation of adding a liquid medium overlay did not reduce STN	Barghchi and Alderson (1996)
*Pistacia vera* L. cv. NR	Optimum SMM: DKW + 5 µM BA + 0.5 µM IBA + 0.01 g/l AA. STN = 25% (DKW), 45% (MS), 60% (WPM)	Use of CG, shoot density in flasks, flask ventilation, flask volume, bottom cooling	Improvements in STN when using bottom cooling (50% STN), reducing shoot number per flask from 7 to 5 (52% STN), or use of ventilated jars with larger volume (58% STN), relative to the control (75% STN) or the addition of 3 mM CG (80% STN)	García et al. (2011)
*Pistacia vera* L. cv. Ohadi, Kalleghochi	SMM: unrooted shoots on MS + 4 mg/l BA + 0.25 mg/l NAA tested for STN after 4–6 weeks; callus production and media browning also observed. STN = UQ	NAA inhibited CK production; callus that formed at base of shoots used nutrients; rooted shoots may absorb nutrients; insufficient Ca^2+^ uptake	No suggestions. STN initially detected in Barghchi and Alderson (1983)	Barghchi and Alderson (1985)
*Platanus acerifolia* (Ait.) Willd	Optimum SMM: MS + 1.33 µM BA + 0.27 µM IAA. STN = 57% (gelled medium), 69% (liquid medium), but wide variation (~ 20–69%) depending on the genotype	Use of liquid medium	Use of solid medium (gelled with 7 g/l agar)	Alegre et al. (2015)
*Populus alba* L. × *P. tremula* L.; *P. trichocarpa* Torr. & A.Gray ex. Hook. × *P. deltoids* W.Bartram ex Marshall	Optimum SMM: WPM + 0.5 mg/l MES + 0.02 mg/l TDZ. STN in transformation experiments = UQ	${\text{NO}}_{3}^{ - }$/${\text{NH}}_{4}^{ + }$ ratio, especially < 0.8 mM ${\text{NH}}_{4}^{ + }$ in medium; medium pH < 4.9; Ca deficiency	Medium without TDZ could not form shoots; after 7 days, ${\text{NH}}_{4}^{ + }$ conc. decreased from 5.0 mM to 1.6 mM; use of 650 mg/l CG + 0.5 mg/l MES + 2.5 µg/l BA allowed shoot growth without STN	De Block (1990)
*Portulaca grandiflora* Hook	Optimum SMM: MS + 4 µM BA + 8 µM Kin. STN = 80–90% (0.1–0.4 mM B); ~ 75–100% (3, 6, 12, 18, 24 and 30 mM CG); 50–85% (3, 6, 9, 12, 24 and 30 mM CaCl_2_)	Insufficient Ca^2+^ and ${\text{BO}}_{3}^{ - }$	Use of 18 mM CaCl_2_ reduced STN to 40%	Srivastava and Joshi (2013)
*Prunus armeniaca* L. cv. Helena, Lorna	Optimum SMM + RIM: QL + 1.78 µM BA + 0.2 µM IBA. STN was observed in the rooting phase (~ 65% for ‘Lorna’; ~ 75% for ‘Helena’)	No reason provided	Adding 0.2 mg/l BA reduced STN to ~ 5% in ‘Lorna’ (~ 25% for ‘Helena’), but this also reduced rooting efficiency. High rooting ability of both cultivars maintained with reduced STN when 5–20 mg/l BA added	Pérez-Tornero and Burgos (2000)
*Prunus armeniaca* Lam	Optimum SMM: WPM + 0.5 mg/l BA. STN = UQ, only weighted	Low ${\text{NH}}_{4}^{ + }$ and ${\text{NO}}_{3}^{ - }$. Low mesos (Ca^2+^, Mg^2+^, K^+^)	Critical threshold for CaCl_2_·2H_2_O: 2.94*x*. If CaCl_2_·2 H_2_O > 2.94*x* interaction with KH_2_PO_4_, so it should be higher than 1.12*x*. Recommended ${\text{NO}}_{3}^{ - }$ level: > 45 mM. Considering STN and other growth factors, optimum range of ${\text{NO}}_{3}^{ - }$ is > 25 mM and ≤ 35 mM and optimum ${\text{NH}}_{4}^{ + }$/Ca^2+^ ratio is ≤ 0.8	Kovalchuk et al. (2017a, b, 2018)
*Pyrus communis* cvs. Old Home × Farmingdale 87, Horner 51, Winter Nelis; *P. dimorphophylla*; *P. ussuriensis* cv. Hang Pa Li	Optimum SMM: MS + 4.44 µM BA. STN = UQ, but genotype-dependent and characterized as a function of significant interactions between multiple factors	General trends: low mesos (Ca Ca^2+^, K^+^, Mg^2+^) and N caused STN. *P. communis*: low mesos + low Fe and N; *P. dimorphophylla*: high NH_4_NO_3_, mesos + Fe with low KNO_3_; *P. ussuriensis:* low NH_4_NO_3_, KNO_3_ and mesos + high Fe and micros caused STN	STN was reduced by increasing the mesos (*P. communis*), using low NH_4_NO_3_, KNO_3_ and high mesos (*P. dimorphophylla*), and using high KNO_3_ and low mesos (*P. ussuriensis*). STN frequently occurred simultaneously with other physiological problems such as callus induction, hyperhydricity, hypertrophy, fasciation and formation of hooked leaves	Reed et al. (2013)
*Pyrus* L. cv. Williams, Highland	Optimum SMM: half-strength MS or WPM + 10 µM BA + 55 µM ABA. After 4 weeks, STN was 23%, 31%, 14% and 14% in MS, ½MS, WPM and QL, respectively, for Williams (30%, 52%, 10% and 64% in Highland)	No substantiated reason provided. Level of STN differed with sampling time (2 vs 4 weeks)	Adjusting medium pH, levels of Ca^2+^, BA or GA_3_ had no visible effect on STN levels in both cultivars. WPM and low levels of Ca^2+^ should be used	Grigoriadou et al. (2000)
*Pyrus* sp. cv Punjab Beauty; rootstocks Patharnakh (*P. pyrifolia* [Burm F.] Nakai), Kainth (*P. pashia* Buch. Ham.), Shiara (*P. serotina* Rehd.); wild pear (*P. pyrifolia*)	Optimum SMM: MS + 4.44 mM BA + 2.46 mM IBA. STN = 79% (Punjab Beauty), 50% (Patharnakh), 25% (Kainth), 18% (Shiara), 7% (wild pear)	Addition of auxins (NAA, IBA), alone or in combination, increased STN in solid or liquid medium, but response was genotype dependent: 38% in control to 40–48% in wild pear, Kainth and Punjab Beauty; 23% in control to 25–30% in Patharnakh and Shiara	Response to Ca^2+^ and ${\text{BO}}_{3}^{ - }$ levels was genotype-dependent on half-strength MS. In wild pear, STN = 9% (control), 3% (3 mM Ca^2+^ + 100 µM ${\text{BO}}_{3}^{ - }$), 56–65% (6 or 9 mM Ca^2+^ + 100 µM ${\text{BO}}_{3}^{ - }$), 0% (1.5 mM Ca^2+^ + 100, 200, 500 or 1000 µM ${\text{BO}}_{3}^{ - }$). Except for 1.5 mM Ca^2+^ + 200 µM ${\text{BO}}_{3}^{ - }$, all other Ca^2+^ and ${\text{BO}}_{3}^{ - }$ treatments increased STN in Punjab Beauty (from 72% in control to 73–82%). In Patharnakh, STN decreased from 42% (control) to 35–36%, but increased to 50% with 9 mM Ca^2+^ + 100 µM ${\text{BO}}_{3}^{ - }$. These Ca^2+^ and ${\text{BO}}_{3}^{ - }$ treatments did not increase or decrease STN in Kainth and Shiara	Thakur and Kanwar (2011)
*Pyrus* sp. rootstocks Pyrodwarf, OHF	Optimum SMM: MS + 2.5 mg/l BA + 0.2 mg/l IBA. STN = 0–68% (OHF), 0–27% (Pyrodwarf) across 27 media with combinations of KNO_3_, NH_4_NO_3_, CaCl_2_, MgSO_4_, and KH_2_PO_4_, and three basal media (MS, QL, WPM)	${\text{SO}}_{4}^{ - }$ ions and ${\text{NO}}_{3}^{ - }$ ions affected incidence of STN the most in OHF and Pyrodwarf, respectively	Neural network modeling and regression analysis were used to assess the severity of STN and to optimize medium components to reduce the incidence of STN. After model optimization, STN estimated to be 0% in OHF and 0.2% in Pyrodwarf	Jamshidi et al. (2016)
*Quercus alba* L., *Q. robur* L., *Q. rubra* L	Optimum SMM: WPM + 0.2 mg/l BA (2 weeks) then 0.1 mg/l BA (4 weeks). STN (*Q. rubra* only) = 22% (genotype 1), 44% (genotype 2), 3–5% (both genotypes on SMM + 3 mg/l AgNO_3_	No reason provided	Addition of 3 mg/l AgNO_3_	Vieitez et al. (2009)
*Rhododendron* ‘P.J.M. Hybrids’; *Rubus* sp. ‘Dirkson Thornless’; *Hibiscus rosa-sinensis* L	Basal medium: MS (Anderson (1975) for rhododendron) + no IBA (rhododendron) or + 10 µM IBA (hibiscus, blackberry). STN: 18% decrease in hibiscus, 39% decrease in rhododendron when 10 µM IBA used; in blackberry, 21% decrease when 1 µM IBA used, or 13% increase when 10 µM IBA used	Production of autoinhibitory exudate with polyphenols from cut surface	Addition of 10 µM IBA for hibiscus and rhododendron (1 µM IBA for blackberry). Objective was not to assess shoot or root growth, only STN (measured as length of shoot tip or stem blackening). In hibiscus, as BA was increased from 0 to 10 µM, STN increased	Compton and Preece (1988)
*Rhododendron* cv. Dopey, Hoppy and Sneezy; *Disanthus cercidifolius* Maxim.; *Crataegus oxyacantha* L. cv. Paul’s Scarlet	Optimum SMM: WPM + 2.5 µM 2iP (*Rhododendron*); LS + ½ MS (macro) + 3 µM BA (*Disanthus*); LS + 2.5 µM BA + 0.5 µM IBA (*Crataegus*). STN = 33–43% (depending on light filter applied) (*Crataegus*); 0% at 11 or 26 µmol m^−2^ s^−1^, 13% at 55 µmol m^−2^ s^−1^, and 64% at 106 µmol m^−2^ s^−1^ (*Disanthus*)	High light intensity (55 or 106 µmol m^−2^ s^−1^); reduced chlorophyll content; photolysis of endogenous auxin (theory)	STN not observed in any of the three *Rhododendron* cultivars, which grew equally well at all light intensities. *Crataegus* and *Disanthus* cultures should be grown at low light intensities (11 or 26 µmol m^−2^ s^−1^)	Marks and Simpson (1999)
*Rosa clinophylla* Thory	Optimum SMM: MS + 28.3 µM AA + 26 µM CA + 58.85 µM AgNO_3_. STN = 80% (control); Kn and AgNO_3_ treatments = UQ	Addition of CK (Kn) at 1.16–4.64 µM to SMM	Addition of 58.85 µM AgNO_3_	Misra and Chakrabarty (2009)
*Rosa hybrida* cv Tineke	Optimum SMM: MS + 2 mg/l BA + 0.5 mg/l NAA. STN = 0% (0 µM ACC), 6% (10 µM ACC), 13% (25 µM ACC), 31% (50 µM ACC), 38% (100 µM ACC) in the absence of IAA; 38% (0 or 10 µM ACC), 56% (25 µM ACC), 69% (50 µM ACC), 81% (100 µM ACC) in the presence of 1 mg/l IAA	Increased ethylene in response to increased levels of ACC, in the presence or absence of IAA	IAA is likely not the most suitable auxin. The use of ethylene-inhibiting compounds (STS, SNP) improved apical shoot initiation (likely eliminated STN by removing ethylene)	Park et al. (2016)
*Rubus idaeus* L. cv. Allgold, Erika, Polka	SMM: unrooted shoot tips on MS + 0.6 mg/l BA after 30 and 60 d. STN = partly quantified	Browning (18–45% of explants in Allgold, 5–63% in Erika, 18–58% in Polka, depending on the medium); Ca deficiency	Lowest explant browning on medium with 1 mg/l CG (following Singha et al. 1990), resulting in 100% shoot initiation and survival (65–90% in controls (no CG), depending on the cultivar). AA at 50 and 100 mg/l reduced explant browning and STN in Allgold and Polka, but increased both phenomena in Erika. Explant browning and shoot initiation negatively correlated (*R* = 0.997); STN was also negatively correlated with shoot survival (*R* = 0.811)	Amalia et al. (2014)
*Salix tarraconensis* Pau ex Font Quer	Optimum SMM: MS + 4.9 µM 2iP. STN (SMM) = 0% in MS or WPM without 2iP; 23–37% (MS + 0.98–9.8 µM 2iP); 50% (WPM + 0.98 µM 2iP). STN (WPM-based rooting medium) = 7% (auxin-free control); 13–27% (1.14–5.71 µM IAA); 27–60% (0.98–4.9 µM IBA); 0–7% (1.07–5.37 µM NAA)	MS medium (relative to WPM). Presence of 2iP in basal medium. Inclusion of IAA and IBA as auxins in rooting medium	Use of a low concentration of 2iP (2.46 or 4.9 µM) in WPM for SMM for low levels of STN (7%). Use of NAA as the auxin for rooting	Amo-Marco and Lledo (1996)
*Solanum tuberosum* L. cv Dark Red Norland	Optimum SMM: MS + 0.5 mM *myo*-inositol. STN = UQ	Low Ca^2+^ content	When Ca^2+^ conc. was increased from 1 µM to 3000 µM, number of axillary shoots decreased from 21 to 1. STN increased when 5 mM EGTA was added to SMM, but decreased when 204 µM strontium was added	Ozgen et al. (2011)
*Solanum tuberosum* L. cv. Burbank, Dark Red Norland, Atlantic, Superior, Snowden	Same as Ahmed and Palta (2017a). STN (Acros/Fischer agar) = 29%/20% (250 μM CaCl_2_), 18%/13% (500 μM CaCl_2_), 0%/0% (2000 μM CaCl_2_) for cv. Atlantic, 29%/21% (250 μM CaCl_2_), 14%/0% (500 μM CaCl_2_), 7%/0% (2000 μM CaCl_2_) for cv. Snowden, 45%/25% (500 μM CaCl_2_), 17%/18% (1000 μM CaCl_2_), 0%/6% (2000 μM CaCl_2_) for cv. Burbank, 34%/23% (500 μM CaCl_2_), 6%/13% (1000 μM CaCl_2_), 0%/0% (2000 μM CaCl_2_) for cv. Superior, 12%/6% (500 μM CaCl_2_), 0%/7% (1000 μM CaCl_2_), 0%/0% (2000 μM CaCl_2_) for cv. Dark Red Norland, at 15 d; 79%/87% (250 μM CaCl_2_), 59%/38% (500 μM CaCl_2_), 6%/0% (2000 μM CaCl_2_) for cv. Atlantic, 29%/28% (250 μM CaCl_2_), 25%/17% (500 μM CaCl_2_), 29%/14% (2000 μM CaCl_2_) for cv. Snowden, 60%/31% (500 μM CaCl_2_), 38%/25% (1000 μM CaCl_2_), 22%/6% (2000 μM CaCl_2_) for cv. Burbank, 44%/33% (500 μM CaCl_2_), 11%/13% (1000 μM CaCl_2_), 12%/0% (2000 μM CaCl_2_) for cv. Superior, 24%/12% (500 μM CaCl_2_), 6%/7% (1000 μM CaCl_2_), 0%/0% (2000 μM CaCl_2_) for cv. Dark Red Norland, at 23 days	Ca deficiency	Inclusion of 250–2000 μM CaCl_2_	Ahmed and Palta (2017b)
*Solanum tuberosum* L. cv. Dark Red Norland	Optimum SIM: MS + 0.56 mM myo-inositol. STN = 56% (60 μM CaCl_2_), 13% (1 μM NAA + 60 μM CaCl_2_), 0% (2 μM NAA + 60 μM CaCl_2_), 52% (250 μM CaCl_2_), 19% (300 μM LPE + 250 μM CaCl_2_), 14% (400 μM LPE + 250 μM CaCl_2_), and 14% (500 μM LPE + 250 μM CaCl_2_) at 15 days; 75% (60 μM CaCl_2_), 23% (1 μM NAA + 60 μM CaCl_2_), 13% (2 μM NAA + 60 μM CaCl_2_), 62% (250 μM CaCl_2_), 29% (300 μM LPE + 250 μM CaCl_2_), 20% (400 μM LPE + 250 μM CaCl_2_), and 24% (500 μM LPE + 250 μM CaCl_2_) at 25 days	Ca deficiency	Inclusion of 300–500 μM LPE (most effective at 400 μM) in Ca-deficient (250 μM CaCl_2_) medium. Inclusion of 1 or 2 μM NAA (most effective at 2 μM) in Ca-deficient (60 μM CaCl_2_) medium	Ahmed and Palta (2017a)
*Solanum tuberosum* L. cv. Russet Burbank, Superior, Norland	Optimum SMM: MS + modified levels of Ca^2+^ (0.3, 3 or 30 mM), as CaCl_2_ or Ca(NO_3_)_2_. STN = 72%, 62% or 48% (Russet Burbank, Superior, Norland, respectively). STN increased from 60 to 100%, 2% to 32%, and 3% to 15% when Parafilm^®^ was used (vs. use of conventional plastic caps) in Russet Burbank, Superior, Norland, respectively	Low Ca^2+^ content. Use of Parafilm^®^	Increasing Ca^2+^ from 0.3 mM to 3 or 30 mM. STN reduced to 0–5%, 4–9% and 1–3% with 3 or 30 mM (Russet Burbank, Superior, Norland, respectively)	Sha et al. (1985)
*Soymida febrifuga* (Roxb.) A. Juss	Optimum SMM: MS + 2 mg/l BA + 0.2 mg/l NAA. STN = 100% (SMM); 7.5% (MS + 556 mg/l CAN + 1 mg/l vit B5); 5.7% (MS + 556 mg/l CAN + 1 mg/l vit B5 + 20 mg/l AC + 100 mg/l fructose); 3.3% (half-strength MS + 556 mg/l CAN + 1 mg/l vit B5 + 20 mg/l AC + 100 mg/l glucose); 1.9% (half-strength MS + 556 mg/l CAN + 1 mg/l vit B5 + 20 mg/l AC + 100 mg/l fructose)	Low Ca^2+^ content. Nutrient content of basal medium	Addition of CAN, vit B5, AC, glucose/fructose, usually together	Chiruvella et al. (2011)
*Trichosanthes dioica* Roxb	Optimum SMM: MS + 37.2 µM Kin. Optimum RIM: half-strength MS + 2.14 µM NAA. STN = 82% (SMM) vs 64% (RIM) at 42 days, but lower in earlier cultures (e.g., 16% (SMM) vs 6% (RIM) at 14 days)	Growth stage (rooting > shoot multiplication)	Supplementing SMM with 0.68 mM CaCl_2_ recovered 93% of shoot cultures with STN (18–38% recovery when 0.34, 1.02 or 1.36 mM CaCl_2_, or 8–24% when 0.32–1.28 mM H_3_BO_3_ was used)	Kishore et al. (2015)
*Ulmus glabra* Huds	Optimum SMM: WPM + 0.4 mg/l BA. STN = UQ	Use of MS (as opposed to WPM)	Use of 0.1 or 0.2 mg/l *m*T	Mirabbasi and Hosseinpour (2014)
*Vitis vinifera* L. cv. Arka Neelamani	Optimum SMM: MS + 1 µM IAA + 0.1 µM GA_3_. STN = 36.3%, but only 1–2 years after initial culture establishment	Cuttings with large leaf area or well developed root system. Choice of explant mass and position. Possibly low availability of Ca^2+^ and Mg^2+^ in shoot tips	STN cultures had higher root: shoot ratio, more roots, and stunted plants than non-STN cultures. STN cultures had Ca^2+^ and Mg^2+^ deficiency in the shoots, but higher levels in the roots, than non-STN cultures. STN ultimately did not negatively impact micropropagation. Solution: selection of explants with medium-sized leaves and density of > 4 plants/vessel	Thomas (2000)
*Vitis vinifera* L. cv. red globe	Optimum SMM: half-strength MS + 1 mg/l BA + 180 mg/l CaCl_2_ + 1.1 mg/l H_3_BO_3_. STN = 39% (2-w subculture), 68% (5-w subculture); 30% (half-strength MS + 1 mg/l BA), 39% (MS + 1 mg/l BA), 48% (half-strength MS + 2 mg/l BA), 51% (MS + 2 mg/l BA), 57% (half-strength MS + 3 mg/l BA), 67% (MS + 3 mg/l BA); (mg/l CaCl_2_ + mg/l H_3_BO_3_): 65% (120 + 1.1), 20% (180 + 1.1), 53% (240 + 1.1), 61% (120 + 2.2), 67% (120 + 3.3)	Low Ca^2+^ (negative correlation between Ca^2+^ content and STN; *R*^2^ = 0.9682). Infrequent subcultures	Adjusting/optimizing the level of BA, and addition of CaCl_2_ and H_3_BO_3_. Frequent (shorter) subcultures. Use of half-strength MS rather than MS	Surakshitha et al. (2019)

Only studies for which data or other evidence was provided are shown; all other studies that claimed to have observed STN, but did not provide evidence or show data are discussed only in the text. Studies for which no data exist to support the claim of STN are not included in this table, but are instead discussed in the main text. Studies listed based on alphabetical botanical name of plant

*2iP* *N*^*6*^-(2-isopentenyl) adenine, *AA* ascorbic acid, *AC* activated charcoal, *ACC* 1-aminocyclopropane-1-carboxylic acid, *AdS* adenine sulfate, *AgNO*_*3*_ silver nitrate, *B* boron, *BA* N^6^-benzyladenine (BA is used throughout even though BAP (6-benzylamino purine) may have been used in the original (Teixeira da Silva 2012), *B5* Gamborg et al. (1968) medium, *Ca* calcium, *CA* citric acid, *CaCl*_*2*_ calcium chloride, *CAN* calcium ammonium nitrate (H_4_CaN_2_O_3_), *CG* calcium gluconate, *CK* cytokinin, *CNT* carbon nanotube, *cv* cultivar, *DKW* Driver and Kuniyuki walnut medium (Driver and Kuniyuki 1984), *EGTA* ethylene glycol tetra acetic acid, *GA*_*3*_ gibberellic acid, *H*_*3*_*BO*_*3*_ boric acid, *IAA* indole-3-acetic acid, *IBA* indole-3-butyric acid, *Kin* kinetin (6-furfurylaminopurine), *LPE* lysophosphatidylethanolamine, *LS* Linsmaier and Skoog (1965) medium, *MES* 2-(*N*-morpholino)ethanesulfonic acid, *mesos* CaCl_2_·2H_2_O, KH_2_PO_4_, MgSO_4_, *Mg* magnesium, *MS* Murashige and Skoog (1962) medium, *mT* *meta*-topolin, *mTR* *meta*-topolin riboside, *NAA* α-naphthaleneacetic acid, *NN* Nitsch and Nitsch (1969), *NR* not reported, *PGR* plant growth regulator, *PVP* polyvinylpyrrolidone, *QL* Quoirin and Lepoivre (1977), *RIM* root induction medium, *s* second(s), *SEM* shoot elongation medium, *SGM* seed germination medium, *SIM* shoot induction medium, *SMM* shoot multiplication medium, *SNP* sodium nitroprusside, *STN* shoot tip necrosis, *STS* silver thiosulfate, *TDZ* thidiazuron (*N*-phenyl-*N*'-1,2,3-thiadiazol-5-ylurea), *UQ* unquantified, *vit* vitamin, *WPM* woody plant medium (Lloyd and McCown 1980), *Zea* zeatin (6-(4-hydroxy-3-methylbut-2-enylamino)purine)

^a^Unlike the majority of other studies where STN was observed after explants were plated or at different stages of in vitro multiplication, in this study, a form of STN was induced as a result of damage induced to shoot tips during explant preparation

De Block (1990) found that STN was linked to Ca deficiency and associated with the use of Woody Plant Medium (WPM; Lloyd and McCown 1980). De Block (1990) also noted that the occurrence of STN might have been caused by a decrease in medium pH, possibly as a result of ammonium (${\text{NH}}_{4}^{ + }$) uptake by shoots. Relative to Murashige and Skoog (1962) (MS) medium, WPM has almost the same Ca^2+^ content (≅ 3 mM in WPM), about a quarter the concentration of ${\text{NH}}_{4}^{ + }$ (20.61 vs 5.00 mM) and nitrate (${\text{NO}}_{3}^{ - }$) (39.41 vs 9.71 mM), about two-thirds the content of K^+^ (20.05 vs 12.61 mM), about a quarter of the ${\text{NH}}_{4}^{ + }$/Ca^2+^ ratio, but more than 1.5-times higher Ca^2+^/K^+^ ratio (Suppl. Table 2). MS was employed in 68.6% of the studies listed in Table 1 while 21.4% used WPM. This suggests that the use of these basal media is not recommended, especially for trees and woody shrubs. This is curious if one considers that WPM was designed specifically for Ericaceous woody plants. The most popular theory for the cause of STN is related to nutrient deficiency and imbalance. Another is the impact and imbalance of PGRs. These possibilities are explored in greater detail next.

## Nutrient deficiencies

### Calcium deficiency

The most commonly ascribed reason for STN is Ca deficiency (32.8% of studies in Table 1). Table 1 indicates that one of the most popular methods to relieve STN has been to increase Ca^2+^ concentration in the culture media (35.9% of studies in Table 1). In pistachio, Barghchi and Alderson (1985) suggested that STN was caused by Ca and boron (B) deficiency, but only on some shoots that had not rooted. Dolcet-Sanjuan and Claveria (1995) reduced STN by lowering the concentration of Ca^2+^ (as calcium chloride, CaCl_2_·2H_2_O) in medium to one-third of the level in MS, and by reducing the subculture period from 4–6 weeks to 3 weeks.

Kovalchuk et al. (2017a) used a CART (classification and regression tree analysis) decision tree to model the incidence of STN in wild apricot (*Prunus armeniaca* L.) shoot cultures based on previous response surface methodology (RSM). They noted that no STN developed in wild apricot shoots when CaCl_2_·2H_2_O was < 2.94 mM, which is the precise concentration of CaCl_2_·2H_2_O in WPM medium (Suppl. Table 2). Furthermore, the Reed et al. (2013) study of pear (one of several connected studies), which was based on MS medium, noted an increase in STN with low mesos (CaCl_2_·2H_2_O, MgSO_4_·7H_2_O, KH_2_PO_4_) but also the involvement of ammonium nitrate. Wang and van Staden (2001) doubled the concentration of CaCl_2_ in WPM to 6 mM to reduce the incidence of STN in tree peony (*Paeonia* × *suffruticosa* Andrews) cultures. Machado et al. (2014) found that the incidence of STN was halved when the level of CaCl_2_·2H_2_O was increased threefold from 440 to 1320 mg/l (from 3.96 mM to 11.89 mM) in true lavender (*Lavandula angustifolia* Mill.) shoot multiplication medium. Christensen et al. (2008) completely eliminated STN in Chinese hibiscus (*Hibiscus rosa-sinensis* L.) shoot cultures after increasing CaCl_2_ concentration in MS from 2.99 mM to 9 mM, independent of the N^6^-benzyladenine (BA) concentration used (0.22 or 2.2 µM). STN was observed in cultures of potato (*Solanum tuberosum* L.) ‘Dark Red Norland’ when insufficient (68 µM) CaCl_2_ was provided, resulting in a loss of apical dominance and enhanced axillary branching, a response that did not occur when there was sufficient (1360 µM) Ca^2+^ in medium (Busse et al. 2008). The level of CaCl_2_ was one of the factors that affected the level of STN in Indian lilac (*Azadirachta indica* A. Juss) cultures (Arora et al. 2010). In potato ‘Dark Red Norland’, Ozgen et al. (2011) ascribed the increase in STN, as a result of low Ca^2+^ levels in medium, to injury of the shoot tip and subsequent loss of apical dominance, thereby stimulating axillary shoot formation. In Indian redwood (*Soymida febrifuga* (Roxb.) A. Juss.) cultures, the simultaneous use of calcium nitrate and calcium pantothenate (vitamin B5) at intermediate concentrations could eliminate the incidence of STN (Chiruvella et al. 2011, 2014). Mubina et al. (2018) eliminated STN by doubling the MS-based levels of CaCl_2_ and KNO_3_ in chickpea (*Cicer arietinum* L.) shoot regeneration medium. Nutrient deficiencies or excesses sensu lato accounted for 9.4% of the studies in Table 1. Thirugnanasampandan et al. (2009) found that an adjustment of CaCl_2_ and MgSO_4_ in sarasaparilla (*Smilax zeylanica* Vent.) shoot regeneration medium prevented STN. In lentil (*Lens culinaris* Medikus), increasing Ca^2+^ (up to 750 mg/l, i.e., 6.75 mM) and BA concentration (0.2–0.6 mg/l, i.e., 0.89–2.66 µM) in MS and B5 (Gamborg et al. 1968) basal media decreased the incidence of STN (Ye et al. 2002). That decision that was based on earlier research by Parh et al. (1998). Wetzstein et al. (1989) noted STN during the acclimatization stage of pecan nut (*Carya illinoensis* (Wangenh.) K. Koch) and not in vitro, reducing its incidence by applying a foliar spray of 0.4% calcium nitrate.

Another way to increase Ca concentration in plant culture medium is using calcium gluconate (6.3% of studies in Table 1), usually from the start of shoot induction or multiplication stages (McCown and Sellmer 1987). The application of Ca-gluconate during in vitro culture of hybrid aspen (*Populus alba* × *Populus tremula*) and poplar (*Populus trichocarpa* × *P. deltoides*) eliminated STN in 50% of the shoots (De Block 1990). However, if 3 mM Ca-gluconate was combined with 0.5 mg/l (2.5 µM) 2-(*N*-morpholino)ethanesulfonic acid (pH 5.8), a buffer, then STN was totally eliminated. This may be because Ca-gluconate uptake into cells has a different pathway, via the glucose uptake system, and this form of Ca^2+^ does not involve the release of toxic chloride if CaCl_2_ is used, allowing medium pH to be stabilized and thus ion exchange and uptake to occur at an optimum pH range of 5.6–5.9 (Pasqua et al. 2002). However, the supply of additional Ca^2+^ via CaCl_2_ can also increase the concentration of chloride (Cl^−^) ions, similar to the use of NaCl, and this may be toxic to plant tissues (McCown and Sellmer 1987). In wild apricot, Pérez-Tornero and Burgos (2000) found that the addition of calcium nitrate or Ca-gluconate decreased the incidence of STN but also lowered rooting ability.

Shoot growth rate may be balanced by Ca^2+^ supply to shoots to avoid STN. This balance might depend on species and cultivars, the concentration of other nutrients in the medium that might modify Ca uptake, as well as the tissue or plant’s developmental stage. The form of Ca^2+^ may also affect STN since the same ion (Ca^2+^) content (Suppl. Table 2) can be supplied by different additives (salts or organic forms), but with different uptake mechanisms (Thor 2019) and thus various effects on STN (Table 1). The organic form has a dual uptake mechanism: (1) after dissociation of the Ca^2+^ ion through the highly regulated Ca^2+^ uptake system which is strongly affected by the culture conditions (pH, relative concentration of other cations and anions, etc.); (2) without dissociation, the organic form of calcium is taken up directly into the cytoplasm via the uptake system but the organic part is under completely different regulation (White and Broadly 2003).

### Boron deficiency

Unlike Ca deficiency, where the effect of STN occurs on younger leaves in the growing meristem and develops basipetally, STN caused by B deficiency (6.3% of studies in Table 1) affects older leaves and spread upwards, or acropetally, as was reported in pistachio (Abousalim and Mantell 1994). Martinelli (1988) indicated the same problem in zebrawood (*Pistacia integerrima* J.L. Stewart ex Brandis) and Mt. Atlas mastic tree (*Pistachia atlantica* Desf.). Similarly, Parfitt and Almehdi (1994) found STN in hybrid pistachio UCB-1 (*P. atlantica* × *P. integerrima*), independent of the basal medium used, suggesting that the condition was not based on nutrients. Abousalim and Mantell (1994) confirmed these findings, noticing STN in *P. vera* cv. Mateur shoot cultures, but partially resolved this by adding calcium (Ca^2+^) or boron (${\text{BO}}_{3}^{ - }$). Barghchi and Alderson (1996) used the same approach (see details in Table 1) but could also reduce STN using liquid medium. There is an interaction between ${\text{BO}}_{3}^{ - }$ and Ca^2+^ uptake (Fox and Albrecht 1958): (1) a high ${\text{BO}}_{3}^{ - }$ concentration can improve the uptake of Ca^2+^; (2) boron helps the movement of Ca^2+^ in plants. However, Abdulnour et al. (2000) described that high ${\text{BO}}_{3}^{ - }$ concentrations could adversely affect Ca^2+^ uptake, even causing toxicity if ${\text{BO}}_{3}^{ - }$ levels were as high as 0.4 mM, as in the case of devil’s claw (*Harpagophytum procumbens* (Burch.) DC. ex Meisn.) (Bairu et al. 2009a). Boron deficiency often appears to occur in in vitro cultures of *Pistachia* species. However, the proper balance of nutrients should be assessed due to their interaction.

### Nitrogen deficiency: nitrogen form and quantity

Grigoriadou et al. (2000) found that the occurrence of STN in pear was cultivar dependent and strongly related to the basal medium used. In their study, the application of Quoirin and Lepoivre medium (1977) resulted in the highest rate of STN (64%) in the case of ‘Highland’, while they observed that most shoots were affected by STN on half-strength MS medium in ‘Williams’ (31%). The former medium contains about a quarter the level of ${\text{NH}}_{4}^{ + }$, a quarter of the ${\text{NH}}_{4}^{ + }$/${\text{NO}}_{3}^{ - }$ ratio, and only about 14% of the ${\text{NH}}_{4}^{ + }$/Ca^2+^ ratio compared to MS medium. However, the rate of STN was only 10% in ‘Highland’ and 14% in ‘Williams’ when shoots were cultured on WPM, in which the ${\text{NH}}_{4}^{ + }$/${\text{NO}}_{3}^{ - }$ ratio is the same as in MS medium but the total level of N and the ${\text{NH}}_{4}^{ + }$/Ca^2+^ ratio is only one-quarter of that in MS. In shoot cultures of wild apricot (Kovalchuk et al. 2017a), the use of RSM showed that some STN occurred in control shoot cultures in WPM. However, the influence of ${\text{NH}}_{4}^{ + }$ and ${\text{NO}}_{3}^{ - }$ was much stronger, i.e., when the concentration of these nutrients was low, STN was higher (Kovalchuk et al. 2017b). Ultimately, the recommended level of ${\text{NO}}_{3}^{ - }$ was > 45 mM (Kovalchuk et al. 2018). Excessive ${\text{NH}}_{4}^{ + }$ and ${\text{NO}}_{3}^{ - }$ in two pear rootstock cultures (12.3 and 13.2 mM for OHF; 22 and 20.9 mM for Pyrodwarf) resulted in STN (Jamshidi et al. 2016). In contrast, a shortage of N in dunns white gum (*Eucalyptus dunnii* Maiden) cultures resulted in STN, and the minimum level of N required was 27.7 g/kg (Oberschelp and Gonçalves 2018). The total N content and/or the ${\text{NH}}_{4}^{ + }$/${\text{NO}}_{3}^{ - }$ ratio differ in several media commonly used for the micropropagation of various plant species (Suppl. Table 2; Phillips and Garda 2019). These can cause variation in the growth and developmental responses of in vitro shoots. From the above results, the occurrence of STN appears to depend mainly on the quantity and form of N, the ${\text{NH}}_{4}^{ + }$/Ca^2+^ ratio, and the quantity of mesos elements [mainly Ca^2+^, magnesium (Mg^2+^) and potassium (K^+^)] in medium (Reed et al. 2016; Kovalchuk et al. 2017a, b).

### Interaction of other ions on STN: the ion-confounding effect

Unlike the above studies, which concluded that one of the main reasons for STN was Ca deficiency, some studies did not show any effect of Ca^2+^ on STN (4.7% of studies in Table 1). When Piagnani et al. (1996) applied CaCl_2_ at 3, 9 or 18 mM, this did not reduce the incidence of STN in two sweet chestnut cultivars. In fact, 18 mM CaCl_2_ reduced rooting. When Grigoriadou et al. (2000) increased the level of Ca^2+^, this did not decrease the incidence of STN in pear. Thomas (2000) observed that the balance of Ca^2+^ and Mg^2+^ ions in roots and shoots was responsible for STN. Unlike the trend in most of these studies, Offord and Tyler (2009) found that the addition of Ca^2+^ to half-strength MS medium almost doubled STN in an endangered Australian shrub, pink pimelea (*Pimelea spicata* R.Br.).

Recently, the implementation of knowledge-based design of experiment (DOE) techniques has been widely used for understanding and improving the performance of complex in vitro systems (for example, Wada et al. 2015; Kovalchuk et al. 2017a). Niedz and Evens (2016) reviewed the greatest advantage of DOE in simultaneously minimizing the quantity of data while maximizing data quality based on considering only low order interactions in multi-factor studies (“hierarchical ordering”) on the basis of “sparsity of effects” wherein just a few factors would drive the system efficiently (Box and Meyer 1986).

The use of DOE by Reed et al. (2013) enabled them to conduct a unique experiment to simultaneously study the effect of all macro- and microelements of MS medium on a wide range of physiological disorders in diverse pear germplasms. They divided mineral nutrients of MS medium into five independent groups with the advantage of reducing the required treatment numbers from 3125 (5^5^) to just 43 treatments. Noticeably, their findings asserted that STN is a genotype-dependent disorder that is affected by an imbalance of nutrients in culture media. Therefore, deficiencies in mesos (CaCl_2_·2H_2_O, MgSO_4_·7H_2_O, and KH_2_PO_4_) or nitrogen (either NH_4_NO_3_ or KNO_3_) commonly contributed to STN. Wada et al. (2013, 2015) followed the same approach to improve the quality of many in vitro pear genotypes by readjusting nutrients in MS medium, such as increasing mesos (CaCl_2_, MgSO_4_, KH_2_PO_4_) with increased nitrogen, to eliminate all physiological disorders. In their studies, STN was more evident with lower Ca^2+^ content than MS-based concentrations although lower concentrations of some mesos, including in the MS medium control, may have accounted for the disorders, although no general trend was observed. In addition to the level of CaCl_2_, Arora et al. (2010) reported that other nutrients, principally Ca(NO_3_)_2_, Na_2_SO_4_, and K_2_SO_4_ in basal MS medium, also affected the level of STN in Indian lilac (*Azadirachta indica* A. Juss.) cultures.

The next challenge of tissue culture studies are ion-confounding problems (Niedz and Evens 2006, 2007), wherein salts are subjected as factors in an experimental design and analysis rather than ions by themselves, whilst ions drive the system. For instance, many authors have frequently tried to alleviate STN in different species by increasing the amount of MS-CaCl_2_ because this unique salt contains the Ca^2+^ ion. CaCl_2_ in MS medium releases 2.99 mM Ca^2+^ plus 6 mM Cl^−^ into solution (Suppl. Table 2). Therefore, it is inconclusive to attribute the problem of STN exclusively to Ca^2+^ deficiency while the role of Cl^−^ is completely overlooked. Numerous examples of this inconclusiveness can be found in the literature (Barghchi and Alderson 1996; Piagnani et al. 1996; Bairu et al. 2009a, 2009b; Ozgen et al. 2011; Machado et al. 2014; Poothong and Reed 2014; Surakshitha et al. 2019). Nevertheless, it has recently been proved that Cl^−^ (> 4.67 mM) has a positive effect on reducing STN symptoms in pistachio (Nezami-Alanagh et al. 2019). To the best of our knowledge, the latter study was the first report of the beneficial effect of Cl^−^ on controlling STN in plants.

Ca-gluconate has been reported as a way to alleviate STN in herbal medicinal plants (Srivastava and Joshi 2013), woody shrubs (Amalia et al. 2014), fruit trees (Abousalim and Mantell 1994; Pérez-Tornero and Burgos 2000), and other trees (De Block 1990; Pasqua et al. 2002). As far as we know, the only report to assess the individual role of the gluconate^−^ ion (C_6_H_11_O_7_^−^) in plant growth and development was Nezami-Alanagh et al. (2017). Using artificial intelligence models, a significant negative influence of gluconate^−^ concentration (range  0.0–6.02 mM) on two growth parameters (shoot length and total fresh weight) during pistachio micropropagation was determined. Thus, we strongly advise to cautiously use gluconate in medium formulations for plant micropropagation. Moreover, we also encourage the use of any method (statistical, response surface methodology, chaid or artificial intelligence) that allows the simultaneous study, on one hand, of the effect of a single ion, and on the other hand, of interactions between several factors.

## Plant growth regulators affect STN

Another popular theory to explain the cause of STN is the effect of the level and type of PGRs in the medium. STN has been linked to the level of PGRs in 23.4% of the studies in Table 1. However, an increase in PGRs may alleviate some nutrient deficiencies (Preece 1995). This fortifies the notion that nutrient deficiency is the major cause of STN. STN in apple (*Malus* × *domestica* Borkh.) was attributed to low endogenous hormone content (Kataeva et al. 1991). According to Kataeva et al. (1991), in the absence of roots, where cytokinins (CKs) are mainly synthesized, endogenous CK concentrations in shoots decrease. This affects the synthesis of auxin in the shoot apical meristem, stimulating STN. In sweet chestnut and oak, the absence of CK (BA) in rooting medium, or the presence of a low concentration of BA, induced STN, although the application of BA to cut ends of shoots prior to rooting increased axillary shoot production (Vieitez et al. 1989). When Piagnani et al. (1996) added 5 µM BA to sweet chestnut shoot tips, STN was eliminated, but a mixture of 5 µM BA and 3 mM CaCl_2_ delayed STN. A CK × Ca^2+^  × ${\text{BO}}_{3}^{ - }$ interaction on STN was observed in grape (*Vitis vinifera* L.) cv. Red Globe where supplementary CaCl_2_ and H_3_BO_3_ were needed to suppress STN, even after the level of BA had been optimized (Surakshitha et al. 2019). Thomas (2000) observed that CK concentration had no signficant effect on STN. Surakshitha et al. (2019) did not observe this effect in grape; instead, the level of STN depended on BA concentration. When BA concentration was increased from 8.9 µM (0% STN) to 17.8 µM, cane apple (*Arbutus unedo* L.) cultures displayed 8.7% STN (Gomes et al. 2010). Pérez et al. (1985) reduced STN in filbert (*Corylus avellana *L.) by adding indole-3-butyric acid (IBA) to medium at a low concentration (10 or 25 µM), or by reducing the period of exposure to IBA. In apricot, dipping shoot tips in a solution of BA (1.78–3.11 µM, depending on the cultivar) prior to culture in rooting medium alleviated STN while kinetin had no effect (Pérez-Tornero and Burgos 2000). The mere presence of 2.5 µM BA in MS medium induced STN in moringa (*Moringa oleifera* Lam.) (Hassanein et al. 2018). In pistachio micropropagation, STN was significantly reduced when BA was added at high concentrations (5.77 < BA < 6.66 µM) to basal media (Nezami-Alanagh et al. 2019).

In contrast, in blackberry (*Rubus* sp. ‘Dirkson Thornless’), rhododendron (*Rhododendron* ‘P.J.M. Hybrids’) and Chinese hibiscus, when Compton and Preece (1988) increased BA concentration to 10 µM, STN increased (details in Table 1). Norton and Norton (1985) also noticed STN in *Gaultheria* sp. and *Rhododendron* sp. (Ericaceae) when any concentration of BA was used, although 17 other Ericaceae species did not show STN. As mentioned above, Podwyszyńska and Goszczyńska (1998) found that when indole-3-acetic acid (IAA) was present in medium, the incidence of STN increased in rooting cultures of dwarf rose (*Rosa gymnocarpa* Nutt. ‘Starina’). Lin et al. (2011) also observed STN in Korean pasque flower (*Pulsatilla koreana*) shoots on MS-based rooting medium containing BA and IAA. Serres et al. (1990) observed STN in American chestnut (*Castanea dentata* [Marsh.] Borkh.) in rooting medium containing IBA, and only the top node was affected, allowing lower axillary shoots to form shoots and thus not influencing explant survival. Bairu et al. (2009a) found that the inclusion of BA increased STN in devil’s claw, even more so when an auxin (IAA) was also added. However, the inclusion of *meta*-topolin (*m*T) or *meta*-topolin riboside (*m*TR; more background in Aremu et al. (2012)) could reduce—but not eliminate—the incidence of STN. Kinetin stimulated STN in *Rosa clinophylla* Thory cultures (Misra and Chakrabarty 2009). In buchu (*Coleonema pulchellum* I.Williams) shoot-inducing cultures, STN only occurred when thidiazuron (TDZ) was applied at 13.6 µM in MS basal medium, or in response to 300 µM casein hydrolysate or mebendazole, 40 µM glutamine, or 40 µM glutamine in combination with 4.5 µM TDZ (Baskaran et al. 2014). STN was also observed in grape ivy (*Cissus rhombifolia* Vahl, syn. *Cissus alata* Jacq.) shoot cultures grown in the presence of 4.5 µM TDZ, but not in response to 4.4 µM BA (Dewir et al. 2018). The use of 2 µM TDZ, or even the lack of TDZ, induced STN in 100% of white saxaul (*Haloxylon persicum* (Bunge ex Boiss and Buhse)) shoot cultures. The latter was also associated with stem fasciation, a common response to high concentrations of TDZ (Dewir et al. 2018). Intermediate concentrations (0.5 or 1 µM) of TDZ reduced the incidence of STN by 10–14% (Kurup et al. 2018). The incidence of STN was reduced when 0.1 or 0.2 mg/l (0.8 µM) *m*T was added to the shoot multiplication medium of Scots elm (*Ulmus glabra* Huds.) shoots (Mirabbasi and Hosseinpour 2014). When Marín et al. (2016) replaced BA with 5 µM *m*T in pistachio shoot culture medium, STN was reduced to 20% of cultures.

The application of 15 mg/l (40.7 µM) adenine sulfate prevented STN in nannaari (*Hemidesmus indicus* (L.) R.Br.) (Nagahatenna and Peiris 2007). When Naaz et al. (2014) added 100 mg/l (271.3 µM) adenine sulfate to BA-supplemented MS medium (WPM resulted in higher levels of STN), STN was reduced to 10% in jambolan (*Syzygium cumini* (L.) Skeels.) shoot cultures.

Several other studies assessed the ability of PGRs to reduce STN. Podwyszyńska and Goszczyńska (1998) significantly reduced the incidence of STN in dwarf rose rooting medium containing IAA by adding 2.5–10 mg/l (14.7–58.8 µM) silver nitrate (AgNO_3_), and by increasing the level of MS-based Ca^2+^ 1.5-fold (increasing the level of MS-based Mg^2+^ twofold was optional). AgNO_3_ is an effective ethylene inhibitor (Purnhauser et al. 1987). Vieitez et al. (2009) reduced the incidence of STN in northern red oak (*Quercus rubra* L.) cultures by supplementing medium with 3 mg/l (17.6 µM) AgNO_3_. Martínez et al. (2017) found that AgNO_3_ at 20 µM reduced the incidence of STN in evergreen oak (*Quercus ilex* L.) cultures. Park et al. (2016) found that the production of ethylene in rose (*Rosa hybrida* cv. Tineke) shoot multiplication medium increased the level of STN. They proved this by applying different levels of an ethylene promoter, 1-aminocyclopropane-1-carboxylic acid (ACC), to medium. Ahmed and Palta (2017a) reduced the incidence of STN in Ca^2+^-deficient (6.7 or 27.75 mg/l (60.3–250 µM) CaCl_2_) potato shoot induction medium by adding 1 or 2 μM NAA, or 300–500 μM lysophosphatidylethanolamine (a phospholipid). Curiously, Ahmed and Palta (2017b) found that agars with different levels of Ca^2+^ significantly affected the level of STN: Acros agar was Ca^2+^ deficient (22.92 mg/l (0.5718 mM)) while Fischer Scientific agar was slightly Ca^2+^ deficient (84.36 mg/l (2.1 mM)) relative to the control (MS Ca level = 3000 μM or 120.23 mg/l). However, supplementation with 27.75–221.96 mg/l (0.25–1.99 mM) CaCl_2_ reduced or eliminated STN in five potato cultivars (see details in Table 1). If auxin is used in excess, especially in juvenile pistachio cultures at the rooting stage, STN may develop (Fig. 2).

The ability of endogenous and exogenously added PGRs to alter the level of STN in response to PGR type and concentration, especially during the rooting phase, suggests their important role in STN. To limit or prevent STN, an adequate level of BA and TDZ should be applied, while the application of *m*T and/or its derivatives may be beneficial. Broadly, altering the type or level of exogenously applied PGRs in plant in vitro cultures might not impact STN exclusively, but might also impact many mechanisms, while different genera or species might respond differently (Cardoso et al. 2018). Auxins should not be used at excessive concentrations while ethylene production should be inhibited as much as possible. Excessive ethylene production in plant in vitro cultures can be avoided by applying auxins at a suitable concentration, by increasing aeration of culture vessels (Kumar et al. 1998), using aerated containers, or it can be inhibited by applying ethylene inhibitors such as AgNO_3_ (Teixeira da Silva 2013).

## Other factors and interactions impacting the incidence of STN

### Timing of measurements and subculture length

Grigoriadou et al. (2000) noted that the level of STN was much higher at 4 weeks than at 2 weeks, suggesting that sampling time influenced the quantitative outcome. This issue was not raised in most other studies on STN but is an important issue to consider when dealing with plant tissue cultures (Teixeira da Silva and Dobránszki 2013). Srivastava and Joshi (2013) found that STN was 62% after 2 weeks, but 90% after 4 weeks in rose moss (*Portulaca grandiflora* Hook.) cultures. The same time-dependent incidence of STN was observed in tissue cultures of five pear cultivars (Thakur and Kanwar 2011). The time-sensitive outcome of STN was also observed by Kishore et al. (2015) in pointed gourd (*Trichosanthes dioica* Roxb. var. Swarna Alaukik). They observed higher STN (83%) during shoot multiplication at 42 days than at 14 (16%), 21 (44%), 28 (61%), and 35 (72%) days on MS medium containing 3% sucrose, 0.8% agar, 0.02% carbendazim and 37.17 µM kinetin. Ahmed and Palta (2017a) observed 56% STN in Ca^2+^-deficient (60 μM CaCl_2_; 52% STN with 250 μM CaCl_2_) shoot induction medium of potato cv. Dark Red Norland when sampled at 15 days, but 75% STN after 25 days (62% STN with 250 μM CaCl_2_). In other words, reported STN levels were higher in older cultures. Similarly, Ahmed and Palta (2017b) found higher levels of STN in the majority of five potato cultivars (i.e., a genotype-specific response) when two Ca^2+^-deficient agar brands were used in shoot induction medium and sampled at 23 days relative to 15 days. Thakur and Kanwar (2011) also observed STN during in vitro rooting on semisolid and liquid medium in five pear cultivars: 6%, 28%, 39%, 49%, and 64% of cultures displayed STN at 14, 21, 28, 35, and 42 days (details in Table 1). Sudha et al. (1998) attributed a long culture period, in excess of 8 months, to the incidence of STN in arka (*Holostemma annulare* (Roxb.) K. Schum.). Amin and Jaiswal (1988) also attributed STN to excessive subculture length in guava (*Psidium guajava* L.) for cv. Chittidar during shoot tip (derived from mature plants) culture on MS medium with 4.4 µM BA. Papadatou et al. (1990), however, did not observe any STN when seedling-derived shoot tips of the same guava cultivar was used on Rugini olive medium (Rugini 1984) with 8.8 µM BA. Delaying the subculture period longer than 2 weeks induced STN in rose and miniature Chinese rose (*Rosa chinensis minima* (Sims) Voss.) (Hsia and Korban 1996). Ca^2+^ concentration that exceeded 6 mM negatively impacted *Pistacia vera* shoot growth and increased shoot chlorosis, but a reduction of the subculture period from 4–5 weeks to 3 weeks reduced the incidence of STN (Dolcet-Sanjuan and Claveria 1995). Tilkat et al. (2008) also found 3 weeks to be suitable for reducing STN in pistachio cultures. Alderson et al. (1987) suggested that increasing the frequency of subcultures, thus reducing the subculture period, could reduce the incidence of STN in dwarf Russian almond (*Prunus tenella* Batsch). A longer subculture length was also associated with hyperhydricity, which is frequently caused by the accumulation of ethylene in cultures (Park et al. 2004).

These results are not surprising. One cause of STN is the deficiency of nutrients, so the chance of nutrient deficiencies within a subculture increases over time as nutrients become depleted (Ramage and Williams 2002). The timing of sampling can influence the reported outcome of STN, although the likelihood of STN is higher in older cultures and may be related to changes in the nutrient content of tissue culture medium over time.

### Genotype-specific responses

Mythili and Thomas (1999) successfully micropropagated two female cultivars (Swarna Alaukik and Swarna Rekha) and one male line of pointed gourd on MS medium but noted a decline in transferable nodes in Swarna Alaukik due to leaf chlorosis if subculture was delayed by 8 weeks. In contrast, no symptoms of STN were observed in pointed gourd accession IIVRPG-102 (Kumar et al. 2016), suggesting that STN could be a genotype-specific response or due to the presence of carbendazim, as was also reported by Kishore et al. (2015). Thakur and Kanwar (2011) observed STN between the 6th and 8th week at the shoot regeneration stage in three pear rootstocks (*P. pyrifolia* [Burm F.] Nakai, *P. pashia* Buch. Ham. and *P. serotina* Rehd.), and two scion cultivars ‘Patharnakh’ (*P. pyrifolia* [Burm F.] Nakai) and ‘Punjab Beauty’ (*P. pyrifolia x P. communis*), but the level of STN was dependent on genotype. Thakur and Kanwar (2011) found a genotype dependence in response to Ca and B supplementation. When 3 µM Ca^2+^ (up from 1.5 µM) and 200 µM ${\text{BO}}_{3}^{ - }$ were used, this completely alleviated the incidence of STN in the wild cultivar (from 9.12% to 2.60%) but had no significant effect nor did it prevent STN in the remaining four cultivars. In London plane tree (*Platanus acerifolia* (Ait.) Willd), Alegre et al. (2015) found a clear influence of genotype on the incidence of STN during shoot multiplication, with a wide range (~ 20–69%) of affected cultures that was genotype dependent. Thus, the susceptibility of a plant to develop STN might be both species and cultivar dependent.

### Choice of basal medium

Bosela and Michler (2008) also noticed that the choice of basal medium affected the level of STN in Eastern black walnut (*Juglans nigra* L.). However, this was also dependent on the in vitro developmental stage and the CK used, with higher levels of STN observed in the presence of Driver and Kuniyuki walnut medium (DKW; Driver and Kuniyuki 1984) and zeatin. Similarly, in unpublished results, STN was observed in vitro cultures of walnut Paradox rootstock during micropropagation in DKW medium (Fig. 2). Shoots were first multiplied on DKW basal medium supplemented with 1 mg/l (4.4 µM) BA, 0.1 mg/l (0.49 µM) IBA and 30 g/l sucrose and subcultured every 3 weeks. Three-week-old shoots, in preparation for rooting, were first placed in the dark for 5 days at 24 °C. STN was observed in rooting medium consisting of DKW free of cytokinins (BA), but including 10 mg/l (44 µM) IBA and 50 mg/l (146 µM) sucrose. After 5 days in rooting medium, auxin-induced shoots were placed in a greenhouse and exposed to high relative humidity (> 95%). These induced shoots rooted and acclimatized concurrently ex vitro.

Curiously, García et al. (2011) observed quite the opposite in pistachio where DKW medium resulted in lower levels of STN than in MS or WPM media. They attributed STN to the three times higher levels of Ca^2+^ in DKW (relative to MS and WPM). Moreover, some authors previously recommended the inclusion of calcium gluconate to prevent STN (Abousalim and Mantell 1994). However, Nezami-Alanagh et al. (2017) fund that gluconate^−^ had an adverse effect on in vitro pistachio plant growth. In high-bush blueberry (*Vaccinium corymbosum* L.), the use of MS medium induced STN, especially when 0.5 mg/l zeatin was used with higher concentrations (> 1 mg/l) of IBA, but when this was replaced by Anderson’s rhododendron medium (Anderson 1984), STN was eliminated (Ružić et al. 2012). Anderson’s rhododendron medium, relative to MS medium, contains about one-quarter the concentration of K^+^, ${\text{NH}}_{4}^{ + }$and ${\text{NO}}_{3}^{ - }$ (Suppl. Table 2). Martin et al. (2007) tested several factors, including PGRs, carbohydrate sources, and AgNO_3_, in the media of subcultured necrotic shoots to try and improve the incidence of STN in in vitro banana (*Musa* spp.) cultures. Normal shoots were recovered only with the addition of 50–100 mg/l (0.45–0.9 mM) of CaCl_2_. When full-strength MS medium was used, STN was observed in *Zeyheria montana* Mart. cultures, but not when half- or quarter-strength MS was used (Cardoso and Teixeira da Silva 2013). Similarly, full-strength MS medium induced STN in Barbados nut (*Jatropha curcas* L.) cultures, but not half-strength MS (Daud et al. 2013), an outcome that Dangi et al. (2014) also observed for bahera (*Terminalia bellerica* (Gaertn.) Roxb.). Using basal CK medium that had diluted levels of MS micro- and macronutrients (Cellárová et al. 1992), Moura (1998) found 15% and 23% STN in shoot initiation and elongation stages, respectively, of leafy St. John’s wort (*Hypericum foliosum* Aiton). The use of WPM induced more STN than MS in the multiplication of wych elm (*Ulmus glabra* Huds.) shoots (Mirabbasi and Hosseinpour 2014). Consequently, the choice of appropriate basal medium can be a solution in itself. Further, altering the level of certain ions, especially Ca^2+^, can also help to reduce STN. However, changing a single medium constituent might affect the uptake or utilization of other nutrients, while agar source and type may affect micronutrients, as discussed elsewhere in this review. Thus, this solution should be viewed cautiously. Moreover, several species responded well to reduced MS salts.

### Antioxidants

Amalia et al. (2014) also noticed some (unquantified) reduction in STN of raspberry (*Rubus idaeus* L.) shoots when 50 or 100 mg/l (0.284–0.568 mM) of ascorbic acid was used, but not as effectively as the use of 1 g/l Ca-gluconate. The reduction in STN was also genotype dependent. Misra et al. (2010) were also able to reduce STN in Barbados nut cultures by adding antioxidants, either 25 mg/l (81.3 µM) of reduced glutathione or 10 mg/l (56.7 µM) of ascorbic acid. Jaiswal et al. (2013) observed STN in Indian kino tree (*Pterocarpus marsupium* Roxb.) cultures. They eliminated STN by adding 568 µM ascorbic acid, 260 µM citric acid, 605 µM ammonium sulfate, and 217 µM adenine sulfate to MS basal medium. By adding 1% activated charcoal to root proliferation medium, Sánchez et al. (1997) reduced the incidence of STN from 89 to 30% in sweet chestnut clone 90,025 and from 38 to 13% in clone Pr5. The addition of antioxidants to basal medium during shoot multiplication might be an effective way to reduce or prevent STN.

### Humidity, aeration, and hyperhydricity: is there a link to STN?

**Fig. 3:**
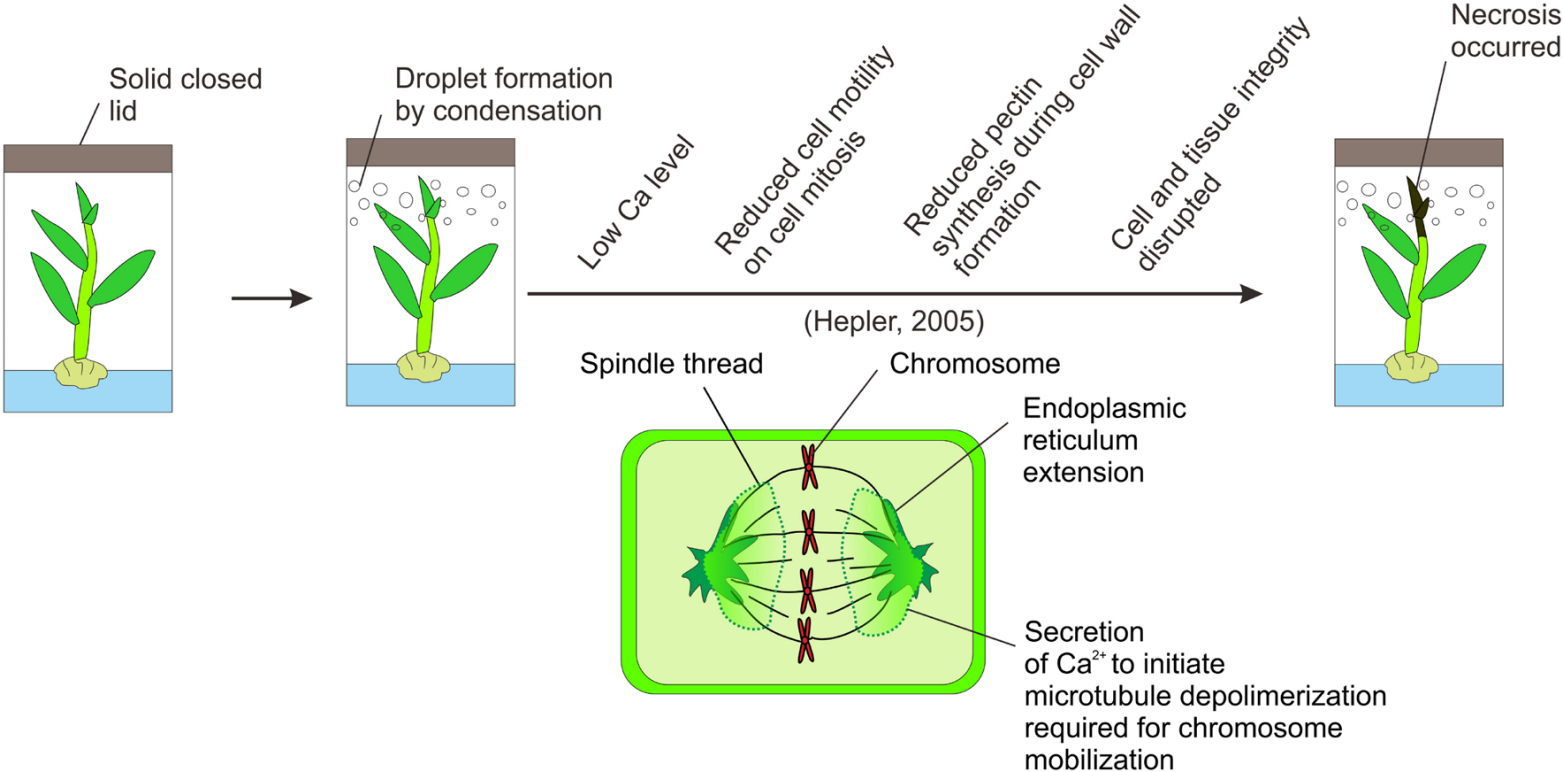
Schematic diagram depicting how high humidity and reduced transpiration in closed tissue culture vessels may induce shoot tip necrosis (STN). Such growth conditions can induce low levels of calcium (Ca) which in turn reduces cell motility and pectin synthesis, disrupting cell (cell wall or cell membrane) and tissue integrity, and reduce transpiration (Hepler 2005), potentially leading to STN. This biochemical hypothesis has still not yet been tested specifically for STN

High humidity and weak ventilation in culture vessels can cause abnormalities, including hyperhydricity (Lai et al. 2005), or STN (Fig. 3a). These abnormalities may in turn be caused by increased ethylene production (Isah 2015). A decrease in humidity within culture vessels can be achieved by improving the ventilation of vessels, or by increasing the agar concentration in basal medium. The former can encourage gas exchange, thereby decreasing ethylene concentration within the vessel (reviewed in Isah 2015). In their summary, Bairu et al. (2009b) concluded that better aeration decreased STN. Barghchi and Alderson (1983, 1985, 1996), in addition to stating that STN was caused by Ca deficiency, also proposed that STN was linked to high humidity in a culture vessel. They found that high humidity reduced plantlet transpiration rate, causing a “low mobility of calcium ions in the xylem”, i.e., reduced nutrient flow to meristematic regions in growing shoot tips. Several authors found that high relative humidity and low transpiration caused by closed culture vessels decreased Ca^2+^ flow during transpiration, causing Ca deficiency (Sha et al. 1985; Singha et al. 1990; Abousalim and Mantell 1994) (Fig. 3a).

Ca^2+^ transport is inhibited by apoplast flooding in which apoplastic air spaces are blocked as a result of water clogging (van den Dries et al. 2013). Bhalla and Mulwa (2003) noted that when Ca^2+^ in medium exceeded 6 mM in macadamia nuts (*Macadamia* F. Muell.), STN symptoms increased. They found that this was as a result of poor culture vessel aeration and high relative humidity and was not linked to Ca^2+^ level in the medium. McCown and Sellmer (1987) suggested that when culture vessels that increase gas exchange or reduce relative humidity are used, hyperhydricity as well as STN were reduced (Fig. 3b), while the use of Gelrite instead of agar improved shoot growth, but increased the incidence of hyperhydricity. Although Matu et al. (2006) did not specifically link aeration problems or hyperhydricity with the incidence of STN in staff tree (*Maytenus senegalensis* (Lam.) Exell) tissue culture, they described this condition as “a major problem”. They improved shoot growth by substituting Gelrite for agar as the gelling agent during shoot multiplication. Offord and Tyler (2009) noted that increased ventilation by employing vented lids for greater transpiration, STN in pink pimelea (*Pimelea spicata* R. Br.) cultures increased from 38 to 73% on MS medium and from 18 to 56% on half-MS medium, but hyperhydricity was observed in both ventilated and unventilated treatments. Compared to cultures on solid medium containing DKW macroelements, MS microelements, 3% sucrose and 0.44 µM BA, cultures of dahlia (*Dahlia x hybrida*) in liquid culture eliminated STN (De Klerk and ter Brugge 2011). Vibha et al. (2014) reached the same conclusion for North Indian rosewood (*Dalbergia sissoo* Roxb.) cultures, reducing hyperhydricity by adding ammonium sulfate to the medium. In quince (*Cydonia oblonga* Mill.), Singha et al. (1990) found that long culture periods and infrequent subcultures resulted in both STN and hyperhydricity, as well as leaf necrosis. However, the application of 3 to 18 mM Ca^2+^ and increasing agar concentration from 0.6% to 1.2% reduced the incidence of these two physiological disorders, but also lowered shoot proliferation and shoot fresh and dry weight. McCown and Sellmer (1987) suggested that some poplar genotypes developed hyperhydricity in response to media with high nitrogen (N) levels. Balla and Kirilla (2006) noted STN in in vitro cultures of peach interspecific rootstocks at the rooting phase. One possible reason was the development of hyperhydricity at temperatures exceeding 22 °C (Balla and Mansvelt 2012). Kataeva et al. (1991) found that the absence of BA in medium resulted in no hyperhydricity, but in high levels of STN, in unrooted apple and tea (*Camellia sinensis* (L.) Kuntze) shoots and in rooted poplar (*Populus tremula* L. × *P. alba* L.) and gerbera (*Gerbera jamesonii* Bolus ex Hooker f.) plantlets (Table 1). However, when BA was added at 4.4 µM into media for apple, hyperhydricity increased to 4% in cotton-covered vessels (18% in foil-covered vessels), even more at 22.1 µM (18% in cotton-covered vessels and 73% in foil-covered vessels), and even more at 22.1 µM with 5.3 µM NAA (58% in cotton-covered vessels and 80% in foil-covered vessels). Had the levels of STN in these four plant species been defined, this would have been an important assessment of the possible link between STN and hyperhydricity.

We recommend the use of culture vessels with improved ventilation and reduced hyperhydricity to reduce the accumulation of ethylene. This would improve Ca^2+^ flow, ultimately reducing the incidence of STN.

## Other factors

Several studies in the literature have reported the incidence of STN in response to factors that are not linked to nutrients, PGRs, or other factors discussed previously.

Lall et al. (1997) observed that exposure of in vitro Mrs Flanagan’s impatiens (*Impatiens flanaganiae* Hemsl.) plantlets to high light intensity (280 µmol m^−2^·s^−1^) for 7 weeks induced necrosis in terminal parts, but it was not clear if this was STN. However, Marks and Simpson (1999) also noticed a similar pattern of increased STN in in vitro cultures of disanthus (*Disanthus cercidifolius* Maxim.) and Northern European hawthorn (*Crataegus oxyacantha* ‘Paul’s Scarlet’), but not of three *Rhododendron* cultivars. In their study, plants were exposed to moderate or high light intensity (55 or 106 µmol m^−2^ s^−1^) and tested against low light intensity (11 or 26 µmol m^−2^ s^−1^), in culture (Table 2).

**Table 2:** Cause–effect (IF–THEN) rules created by neurofuzzy logic indicating the best combination of inputs to alleviate STN in pistachio in vitro cultures

Rules				Membership degree
		SubModel:1		
1	IF	EDTA^−^ is low and K^+^ is low	THEN	Low (1.00)
2		EDTA^−^ is low and K^+^ is high		Low (1.00)
3		EDTA^−^ is mid and K^+^ is low		Low (1.00)
4		EDTA^**−**^ is mid and K^+^ is high		Low (1.00)
5		EDTA^−^ is high and K^+^ is low		High (1.00)
6		EDTA^**−**^ is high and K^+^ is high		High (1.00)
		SubModel:2		
7	IF	BA is low	THEN	High (1.00)
8		BA is high		Low (1.00)
		SubModel:3		
9	IF	IF Cl^−^ is low	THEN	High (1.00)
10		IF Cl^−^ is high		Low (1.00)
		SubModel:4		
11	IF	Genotype is Ghazvini	THEN	High (0.55)
12		Genotype is UCB-1		Low (0.93)
		SubModel:5		
13	IF	Na^+^ is low	THEN	Low (0.78)
14		Na^+^ is mid		High (0.94)
15		Na^+^ is high		Low (1.00)

Inputs with stronger effects have been highlighted by software (for additional details see Nezami-Alanagh et al. 2019)

In American chestnut genotypes B’ville, Iowa #2 and VDW, wounding of cuttings did not significantly affect the rate of STN, but promoted rooting, although the level of STN and rooting was intricately dependent on the level of auxin and cytokinin (Xing et al. 1997). Khalafalla and Daffalla (2008) found that scion length and rootstock age impacted the incidence of STN in grafted gum arabic (*Acacia senegal* (L.) Willd.) shoot tips. They registered 7% STN when scions were 2.5–3 cm, 27% when they were 1.5–2 cm, or 14% when rootstocks were 7 days old (0% STN when rootstocks were 14 days old). The incidence of STN in grape cv. Arka Neelamani also depended on the position of the explant on the stock plantlet and its initial weight (Thomas 2000).

The establishment of STN is also influenced by light intensity during shoot multiplication, and other factors such as rootstock age.

## Possible mechanisms underlying STN

### Programmed cell death, necrosis, and stress

It is possible that the underlying mechanisms to explain explant wounding and subsequent tissue browning may be similar. However, since we consider STN to be a physiological response of a living tissue on a developing in vitro plant, rather than a cut explant, we will hereafter only consider the possible factors that might affect STN. The use of chemicals such as antioxidants (e.g., Misra et al. 2010), reduction in light intensity since high light intensity stimulates polyphenol oxidase (Krishna et al. 2008), or the inhibition of phenylpropanoid biosynthesis (Jones and Saxena 2013) may be viable ways to alleviate STN, similar to tissue browning after explant cutting during the establisment of an in vitro culture. When oxidative stress can no longer be controlled, programmed cell death (PCD) develops (Gaspar et al. 2002), which may explain STN. Beckman (2000) further suggested that specialized cells induced cell suberization and lignification as a result of the accumulation of phenolic compounds, thereby strategically stimulating PCD. PCD develops as a function of H_2_O_2_, the “death signal”, and in response to other reactive oxygen species (Demidchik 2015). PCD affects several developmental events in plants, including sensescence where proteins, phospholipids and pigments may be degraded (Drury and Gallois 2006; Henmi et al. 2007; Misra et al. 2010; Kacprzyk et al. 2011). Ethylene, which can accumulate in closed tissue culture vessels in vitro, is a strong inducer of leaf senescence and a trigger for PCD (Santner et al. 2009; Trobacher 2009; Park et al. 2016). Moreover, ethylene initiates a signaling pathway, including calcium transport, during the development of aerenchyma, which also plays a role in PCD (Jones 2001). It is still unclear if PCD is involved in, related to, or the cause of STN.

### Calcium and calcium signaling

**Fig. 4:**
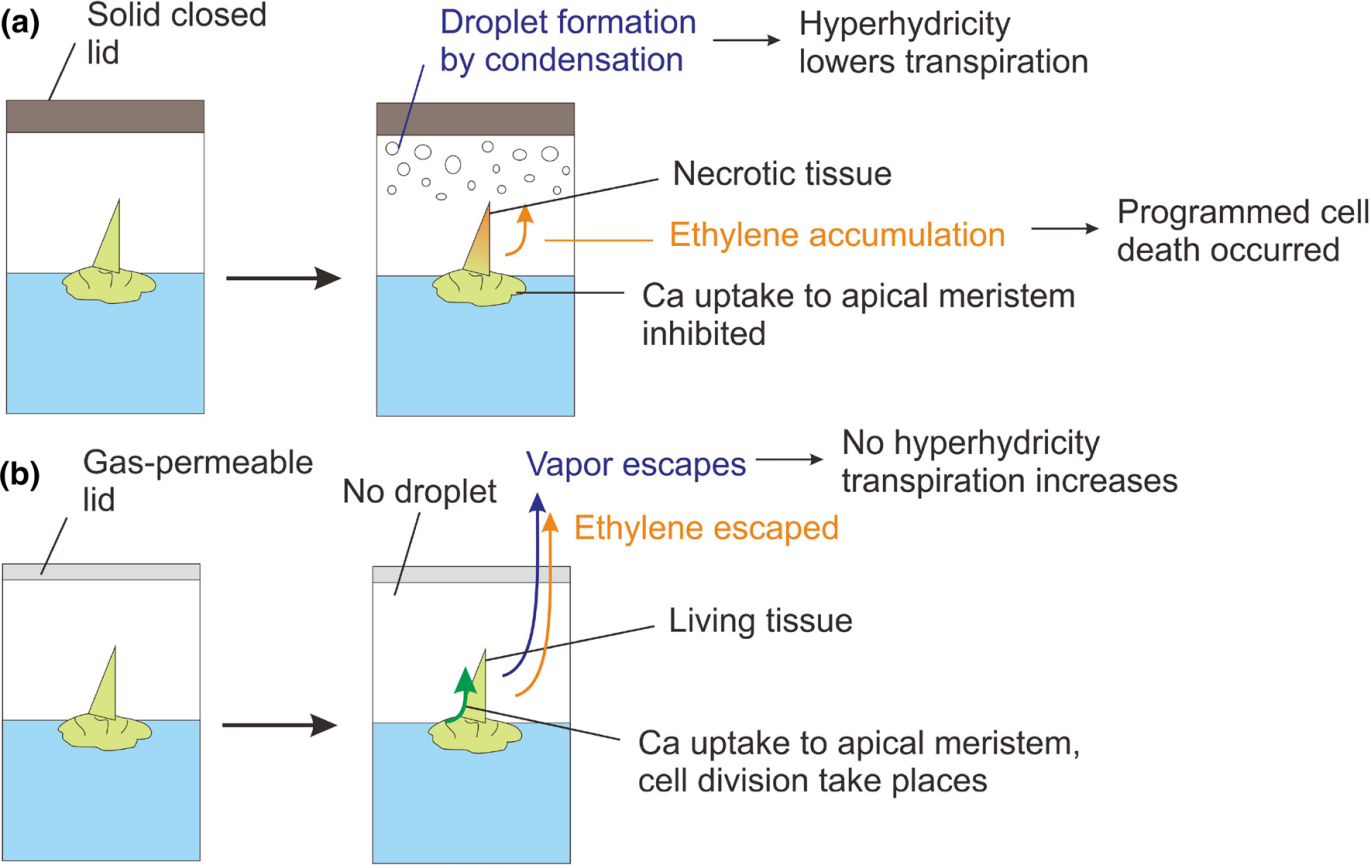
The impact of hyperhydricity, as a result of poorly ventilated culture vessels, may promote shoot tip necrosis (STN). Schematic representation of the possible mechanisms involved in STN when an in vitro plant culture is established in a closed vessel, causing ethylene to accumulate (**a**). Schematic representation of the possible methods to reduce hyperhydricity and ethylene accumulation, by employing gas-permeable culture vessels, to ensure the healthy growth of shoot tips in vitro by reducing or eliminating the incidence of STN (**b**)

Ca deficiency is one of the most commonly cited reasons for STN (Table 1). In closed tissue culture vessels, the high relative humidity and reduced transpiration induced low Ca^2+^ levels, because Ca^2+^ cannot translocate but must be actively transported (Hepler 2005). This does not permit pectin to be synthesized, impeding the formation of the shoot meristem due to compromised cell integrity and membrane permeability (Martin et al. 2007; Naaz et al. 2014) (Fig. 4). Moreover, Ca^2+^ serves as a universal secondary messenger in cellular signaling in plants, so the hormonal balance and Ca^2+^ supply during growth and development may affect each other. There is a correlation between Ca^2+^ and auxin signaling: auxin induces Ca^2+^ signals and vice versa, and Ca^2+^ controls the speed of transport of an auxin (Vanneste and Friml 2013). A high level of auxin might cause excessive ethylene production in jars and change the CK: auxin ratio. As was observed by Busse et al. (2008), STN in potato cultures, which resulted in a loss of apical dominance, was caused by low levels of Ca^2+^ in medium (Ozgen et al. 2011). This theory was confirmed by two experiments (Ozgen et al. 2011): the addition of a Ca^2+^ chelator, ethylene glycol tetra acetic acid (EGTA), to medium with sufficient Ca^2+^ (2720 µM) induced the precise same symptoms as low Ca^2+^ levels, namely STN and axillary shoot formation; in that condition, the supplemental addition of 204 µM strontium (Sr^2+^), which is a Ca^2+^ analog, restored apical dominance. Increasing Ca^2+^ in medium of several tree species has been shown to alleviate STN (McCown and Sellmer 1987). Ca-gluconate is an organic form of Ca that allows Ca^2+^ to be released into aqueous solutions, explaining why it has occasionally been used to alleviate STN, but it negatively affects shoot growth (Singha et al. 1990; Amalia et al. 2014). We suspect that the use of gas-permeable culture vessels, such as the Vitron or Miracle Pack (Teixeira da Silva et al. 2006), could reduce hyperhydricity, reduce the accumulation of ethylene, increase transpiration and consequently increase the transport of Ca^2+^ to the shoot apical meristem, although this hypothesis has yet to be tested on in vitro plant cultures displaying STN.

Since the level of the endogenously accessible Ca^2+^ depends not only on its content in the medium but also on its uptake, it is reasonable to expect that the content of other ions in the medium such as Mg^2+^, K^+^, some microelements or ${\text{NH}}_{4}^{ + }$ , which can modify the uptake of Ca^2+^ from the medium based on nutrient interactions (Fageria 2001), may have an effect on STN. In this sense, Ca deficiency may be relative. The content of mesoelements such as Ca^2+^, Mg^2+^ and K^+^ in the medium modified the rate of the STN in different plant species (Reed et al. 2016; Kovalchuk et al. 2017a, b; details in Table 1). In wild apricot shoot culture, the ${\text{NH}}_{4}^{ + }$/Ca^2+^ ratio should be optimally below 0.8, to minimize STN, specifically ${\text{NO}}_{3}^{ - }$  > 45 mM and 25 mM < ${\text{NH}}_{3}^{ - }$  ≤ 45 mM + ${\text{NH}}_{4}^{ + }$/Ca^2+^  ≤ 0.8 for node 5 (Kovalchuk et al. 2018). In shoot cultures of different pear species, besides the role of various mesoelements like Ca^2+^, Mg^2+^, and K^+^, the roles of Fe^2+^ and the proper concentrations of nitrogen compounds were reported to be involved in the occurrence of STN (Reed et al. 2013, details in Table 1).

In addition, Subbaiah et al. (2000) found that activation of protease, which played a role in PCD induced by anoxia in maize (*Zea mays* L.) roots, was Ca^2+^ dependent. The role of Ca^2+^ in plant stress response and signaling has been detailed in a review by Robertson (2013). Bairu (2008) found that the main problem related to Ca deficiency was not the level of Ca^2+^ in medium but its limited transport in plantlets due to excess ${\text{BO}}_{3}^{ - }$. Moreover, Ca^2+^ transport in plants through the xylem sap requires transpiration, which is inhibited by high humidity in the culture vessel, thus the limited mobility of Ca^2+^ can play a role in the development of STN (Hirschi 2004).

### Plant growth regulators

As indicated above, ethylene is a likely inducer of PCD and thus may be a direct cause of STN in unventilated culture vessels. Table 1 indicates that PGRs have been heavily implicated in STN, mostly CKs during the shoot induction stage, but also the CK × auxin interaction during the root induction stage of shoots. For example, the absence or use of low concentrations of CKs was implicated as a reason for the presence of STN since roots are the main source of CKs (Chen et al. 1985), reducing cell division in the shoot apical meristem (Piagnani et al. 1996). The damage to shoot tips reduces the synthesis of auxin because shoot tips are the main site of auxin biosynthesis (Leopold 1975; Aloni et al. 2003; Hopkins and Hüner 2009). Exogenously added CKs can act with different efficiency depending on their structure, the plant species or even the cultivar (Dobránszki and Teixeira da Silva 2010). Application of the highly active *m*T or *m*TR (hydroxylated BA derivatives) can delay senescence and can eliminate abnormalities of in vitro cultures, including a reduction of hyperhydricity and STN (Aremu et al. 2012). Similarly, Kumari et al. (2017) found that when *m*TR was used in the shoot regeneration medium of dwarf wild begonia (*Begonia homonyma* Steud.), the occurrence of shoot necrosis was reduced to about a half of other regenerants cultured on medium with BA or TDZ. After subculture of regenerated shoots onto elongation medium, STN occurred again at a low frequency (18%) if previously regenerants had developed on medium with *m*TR. Bairu et al. (2011) studied the background effects of CK on STN, including an analysis of both endogenous and exogenous CKs and their derivatives, in devil’s claw. They found a higher content of total CKs in necrotic shoots than in normal shoots in all studied cases. However, they also detected larger quantities of deactivated forms of CKs such as 9-glucolides in BA-treated and necrotic shoots relative to normal and *m*T-treated shoots, suggesting that the occurrence of STN may be due to a change of active CKs to other deactivated products, possibly reversibly, but that can be toxic. N^7^ and N^9^ conjugates, which are the inactive forms of BA, are biologically inactive and chemically quite stable, but their conjugation is not fully irreversible (Werbrouck et al. 1996). These conjugates usually accumulate at the base of in vitro shoots, so the active form can be continuously released and cause disorders such as STN (Werbrouck et al. 1996; Strnad et al. 1997). Topolins are hydroxylated forms of BA with high activity in plant tissue culture but they have a different metabolism from that of BA and, therefore, side-effects caused by the release of the active form from inactive BA conjugates can be avoided (reviewed in: Dobránszki and Teixeira da Silva 2010).

## Unmasking the effect of media ingredients on STN using artificial intelligence

In the 1990s, a wide range of statistical methods for the multivariate analysis of plant cell tissue data were employed, but those studies have some limitations (Gago et al. 2010; Gallego et al. 2011): (i) limited kind of data (qualitative or quantitative) can be analyzed using multivariate analysis, but not nominal or image data; (ii) limited application of linear tools such as ANOVA and regression, since biological responses present a high degree of intra- and inter-individual variation that interacts in a non-linear and non-deterministic way; and (iii) a slump in the use of statistical methods to predict or optimize plant tissue culture. In recent years, several parametric approaches such as response surface methodology (RSM), decision trees, Chi-square automatic interaction detector (CHAID), adaptive regression splines and artificial intelligence tools, based on machine learning systems, have been successfully applied to the design of plant tissue media as advanced techniques (Poothong and Reed 2014; Akin et al. 2016,2020). Other computer-based tools based on artificial intelligence tools for understanding the effect of media components on in vitro cultured plants were also explored (Gago et al. 2010; Gallego et al. 2011; Zielińska and Kępczyńska 2013).

**Fig. 5:**
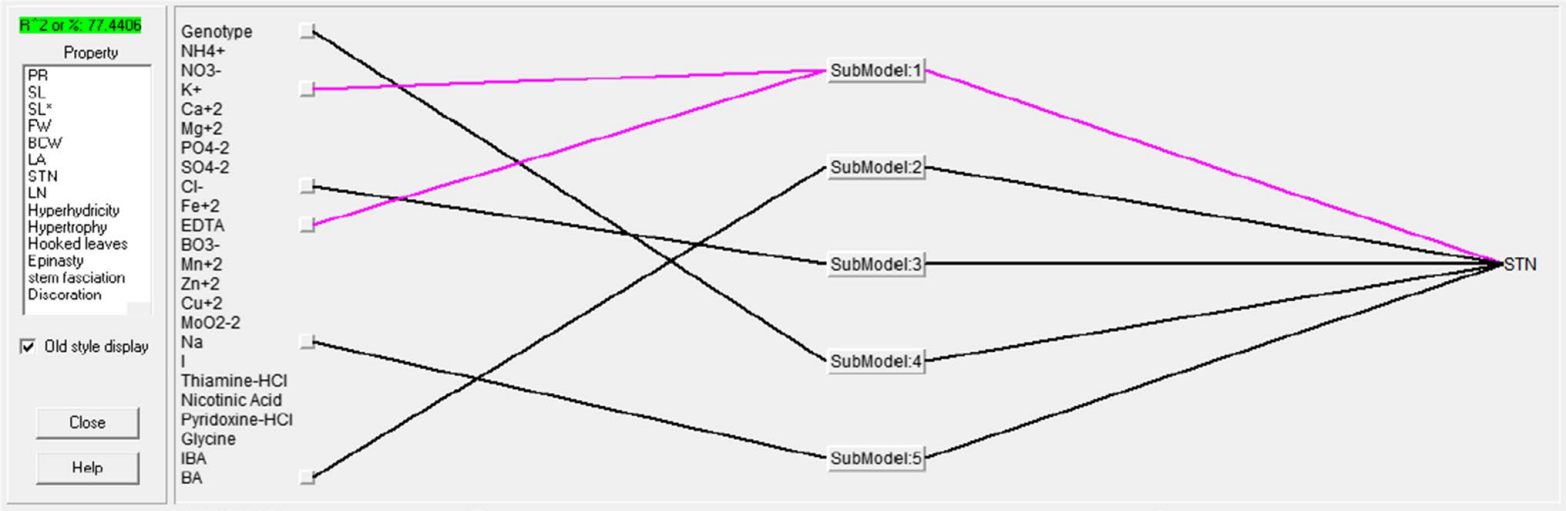
Graphical representation of critical factors affecting STN extracted by neurofuzzy logic. The inputs with stronger effects are marked in pink

Gallego et al. (2011) extensively reviewed the advantages of using artificial neural networks (ANNs) and fuzzy logic, to discover the cause–effect function of various factors on the response of in vitro plantlets. These artificial intelligence tools help researchers to obtain insight of the cause–effect relationships between factors studied (i.e., mineral nutrients) and a wide range of responses (i.e., growth parameters, physiological disorders, etc.). More recently, the combination of DOE with neurofuzzy logic provided them with a powerful tool to obtain a deeper understanding about the effects of culture media composition on different growth parameters (Nezami-Alanagh et al. 2018) and also several physiological disorders, including STN, during micropropagation of two pistachio rootstocks, UCB-1 and Ghazvini (Nezami-Alanagh et al. 2019). Noticeably, in the latter study, STN was successfully modeled with the help of neurofuzzy logic tools, being affected by complex interactions of ions, cytokinin (BA), and genotype. Those results indicated that in pistachio in vitro cultures, STN is caused by the effect of several factors (individually or in interaction), splitting these effects into five sub-models: (1) the complex interaction of ethylenediaminetetraacetic acid (EDTA^−^) × K^+^ as the strongest effect, followed by (2) BA, (3) Cl^−^, (4) genotype and (5) Na^+^ (Fig. 5). The cause–effect induced by STN-related “factors” can be easily understood by several ‘IF–THEN’ rules presented in Table 3, which can be summarized as follows: (i) the strongest effect of low-mid concentrations of EDTA^−^ (0.06 <  ×  < 0.39 mM), regardless of the K^+^ content (rules 5–6). Furthermore, the lowest STN values are also obtained on media including high amounts of BA (1.30 <  ×  < 1.50 mg/l, i.e., 5.72 <  ×  < 6.60 µM) and Cl^−^ (9.10 <  ×  < 17.96 mM (rules 8 and 10). ‘UCB-1′ shoots were more resistant than ‘Ghazvini’ rootstock with respect to STN (rules 11–12). Finally, Na^+^ influenced the appearance of STN in pistachio cultures, with lowest STN when a high concentration of Na^+^ is included in the medium (rule 15).

In conclusion, these findings assert the importance of applying computer-based tools in order to: (i) create a well-sampled design space with the advantage of saving time and costs, (ii) the possibility of splitting salts to their fundamental ions without ion-confounding concerns, (iii) discovery of key factors that impact the measured parameters, and iv) optimization of new cost-effective culture media for healthy in vitro plant growth.

## Conclusion

The literature on STN in in vitro plant micropropagation exceeds 100 studies. While many authors observed STN, in several cases finding practical solutions to eliminate this physiological condition (64 studies in Table 1), many other studies reported the presence of STN, or a reduction in STN, without providing any exact data. Readers should note that Table 1 and Suppl. Table 1 do not reflect the total of all studies that claimed the existence of STN, only those studies that provided quantitative evidence of this phenomenon. In addition, it is likely that many more studies in the plant tissue culture literature may have observed STN. However, STN tends to be observed as a negative finding, but may have either been referred to in other terms, or not reported at all because the publication of negative results is generally not encouraged in mainstream, including plant science, journals (Teixeira da Silva 2015). Thus, the values that we report may underrepresent the true extent of the occurrence of STN.

In summary, first and foremost, the in vitro factor with the most notable influence is nutrient deficiency, mainly Ca, B and N, although there is an interaction with other ions, the ion-confounding effect. The level and type of PGRs in a medium can also impact STN. Other factors that were found to induce STN were the timing of measurements and length of subculture, genotype, choice of basal medium, antioxidants, and possibly humidity, aeration and hyperhydricity.

**Fig. 6:**
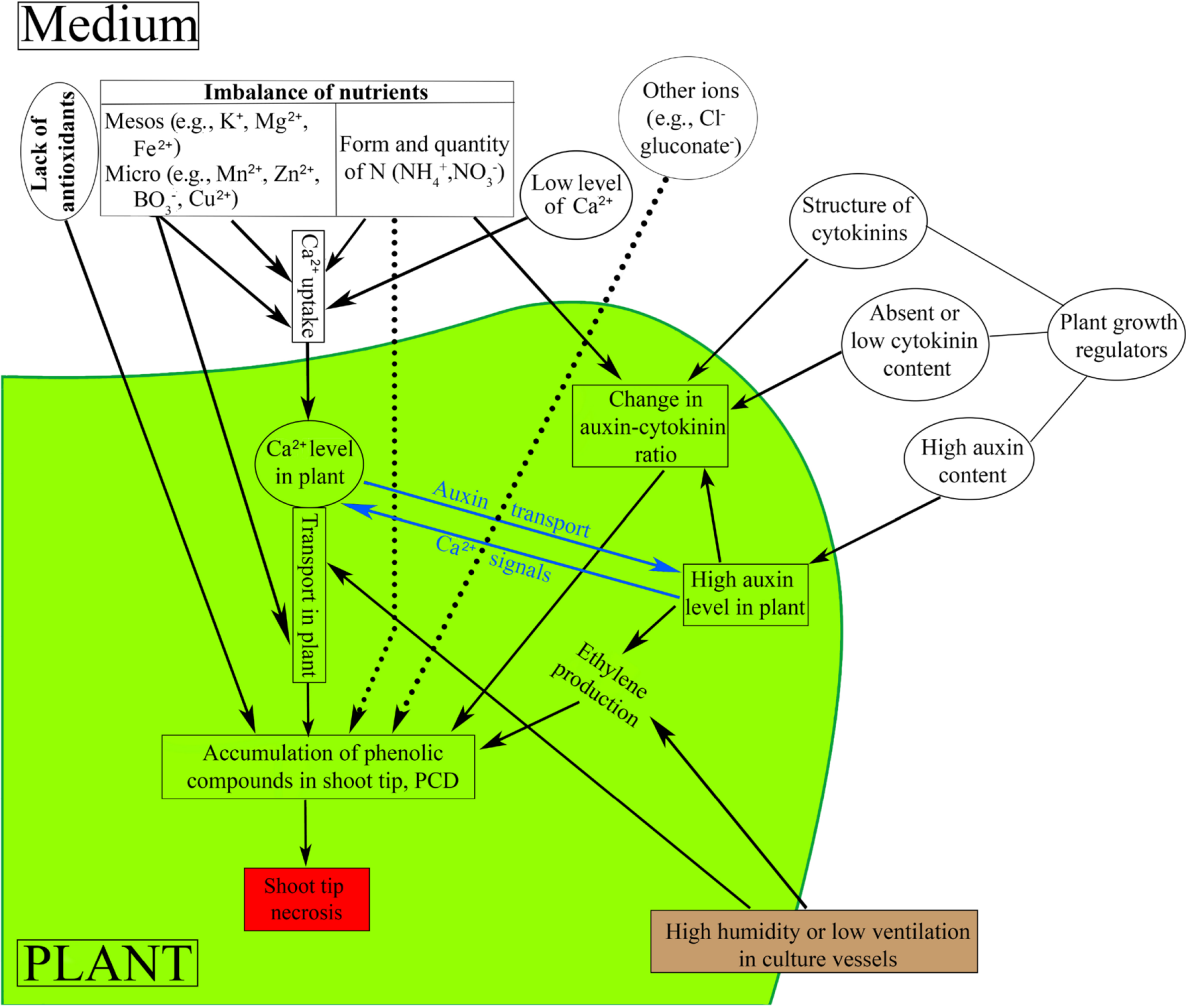
Schematic diagram showing the overall mechanisms of shoot tip necrosis (STN) in in vitro plants. A detailed description may be found in the text, in "Conclusion"

In Fig. 6, we summarized the mechanisms by which STN can occur based on our current knowledge. The lack or imbalance of different nutrients, inappropriate PGR content, and the lack of antioxidants in medium, as well as high humidity and/or low ventillation in culture vessels are proven causes of STN mainly by affecting the uptake and transport of Ca from medium, and modifying the endogenous hormonal balance of in vitro plantlets. Ca deficiency in plants can occur either directly, if a low level of Ca^2+^ is added to the culture medium, or indirectly due to the presence and concentration of other nutrients by modifying Ca uptake based on nutrient interactions. Ca^2+^ can activate polyphenol oxidase (PPO) by changing its conformational state (Ruiz et al. 2003) and peroxidase (POD) by inducing cross-linking in the chains of polygalacturonan (Penel et al. 1999). Thus, Ca^2+^ can inhibit the accumulation of phenolic compounds by stimulating their oxidation and, as a result, it can decrease or inhibit STN, hindering the accumulation of phenolic coumpounds and PCD (continuous arrows). Boron acts in two ways when inhibiting STN. A high concentration of ${\text{BO}}_{3}^{ - }$ can stimulate the uptake of Ca^2+^ and helps Ca^2+^ movement within the plant (continuous arrows). The quantity and form of N (${\text{NH}}_{4}^{ + }$ or ${\text{NO}}_{3}^{ - }$), as well as the ratios of ${\text{NH}}_{4}^{ + }$/${\text{NO}}_{3}^{ - }$ and ${\text{NH}}_{4}^{ + }$/Ca^2+^ affect the occurrence of STN, likely in part through the modification of the ratio of cytokinins to auxins (dotted arrows). The levels of Cl^−^ and gluconate^−^ in the medium can affect the level of STN as well, but the mechanisms by which they act are unknown (dotted arrows). The level and type of PGRs added to the medium also affect the occurrence of STN by changing the levels of ethylene, cytokinins, and auxin. The structure of cytokinins added to the culture medium affects the quantity of their active forms and thus the cytokinin to auxin ratio within the plant. A high level of auxin in a plant modifies Ca^2+^ signaling, while a modified level of Ca^2+^ in a plant modifies auxin transport (blue arrows). Moreover, a high level of auxin in a plant can act directly and generate the production of ethylene, thereby increasing STN. The addition of different antioxidants into the medium can prevent the accumulation of ethylene, thus inducing STN. If relative humidity is high, or if there is weak or no ventilation in culture vessels, this can lead to increased ethylene production and thus STN. High humidity also modifies Ca^2+^ transport within a plant causing its low mobility in the xylem and leading to Ca deficiency in the shoot tip (continuous arrows).

Several possible solutions to eliminating or reducing STN in vitro have been proposed, based on the main factors known for causing STN in vitro (Fig. 6). Despite these proposals, the mechanism(s) remains inconclusive, unexplored, and far from resolved, especially given the wide-ranging, and sometimes contradictory, responses and frequently observed genotype dependence. This review provides, despite not fully understanding the mechanism underlying STN, a more concise and updated summary of studies in STN in vitro with the hope that it will generate new ideas that would allow plant physiologists and molecular biologists to further explore the physiological and genetic mechanisms underlying this disorder.

### *Author contribution statement*

JATdS and MMK conceived the idea and compiled the literature. JATdS wrote the first drafts. In subsequent versions and drafts, all authors contributed equally to ideas, writing, figures, supplements, revisions and corrections. All authors approve the published version and take responsibility for its content.

## Electronic supplementary material

Below is the link to the electronic supplementary material.

Supplementary file1 (DOC 153 kb)

Supplementary file2 (DOC 167 kb)
