# Supplementary material for: Shoot tip necrosis of in vitro plant cultures: a reappraisal of possible causes and solutions
Source: Planta. 2020 Sep 3;252(3):47. doi: 10.1007/s00425-020-03449-4 (PMC7471112; doi:10.1007/s00425-020-03449-4)
Supplement: Supplementary file 1 — Supplementary file1 (DOC 153 kb) [file 425_2020_3449_MOESM1_ESM.doc]

**Supplementary Table 1** Factors, in addition to those listed in Table 1, of plants in which shoot tip necrosis was observed *in vitro* (order of entries based on alphabetical listing of common name)

| **Common name** | **Family** | **Growth habit** | **Main uses/importance** | **Reference** |
| --- | --- | --- | --- | --- |
| American chestnut | Fagaceae | Tree | Nuts; wood | Xing et al. 1997 |
| Apple | Rosaceae | Tree | Fruit | Kataeva et al. 1991 |
| Armenian plum | Rosaceae | Tree | Fruit | Kovalchuk et al. 2017b, 2018 |
| Banana and plantains | Musaceae | Tree | Fruit, food | Martin et al. 2007 |
| Black walnut | Juglandaceae | Tree | Nuts; wood | Bosela and Michler 2008 |
| Blackberry | Rosaceae | Tree | Berries | Compton and Preece 1988 |
| Blue honeysuckle | Caprifoliaceae | Woody shrub | Fruit | Karhu 1997 |
| Chestnut | Fagaceae | Tree | Nuts; wood | Piagnani et al. 1996 |
| Chestnut | Fagaceae | Tree | Nuts; wood | Vieitez et al. 1989 |
| Chinese hibiscus | Malvaceae | Tree | Ornamental; medicinal | Compton and Preece 1988 |
| Chinese hibiscus | Malvaceae | Tree | Ornamental; medicinal | Christensen et al. 2008 |
| Creeping snowberry | Ericaceae | Woody shrub | Ornamental | Norton and Norton 1985 |
| Cymbidium | Orchidaceae | Herbaceous plant | Ornamental | Guha and Usha Rao 2012 |
| Devil's claw | Pedaliaceae | Herbaceous plant | Medicinal | Bairu et al. 2009a |
| Devil's claw | Pedaliaceae | Herbaceous plant | Medicinal | Jain et al. 2009 |
| Devil's claw | Pedaliaceae | Herbaceous plant | Medicinal | Bairu et al. 2011 |
| Devil's claw | Pedaliaceae | Herbaceous plant | Medicinal | Lišková et al. 2016 |
| Dipterocarpus | Dipterocarpaceae | Tree | Wood; reforestation | Linington 1991 |
| Disanthus | Hamamelidaceae | Woody shrub | Ornamental | Marks and Simpson 1999 |
| Dwarf rose | Rosaceae | Woody shrub | Ornamental | Podwyszyńska and Goszczyńska 1998 |
| Ensete | Musaceae | Tree | Fruit; traditional medicine | Diro and van Staden 2005 |
| Filbert | Betulaceae | Tree | Nuts | Pérez et al. 1985 |
| Flame of the forest | Leguminosae* | Tree | Ornamental | Kulkarni and D’Souza 2000 |
| Gerbera | Asteraceae | Herbaceous plant | Ornamental; landscaping | Kataeva et al. 1991 |
| Grape | Vitaceae | Woody shrub | Beverage, fruit | Thomas 2000 |
| Grape | Vitaceae | Woody shrub | Beverage, fruit | Surakshitha et al. 2019 |
| Hawthorn | Rosaceae | Woody shrub | Medicinal; ornamental | Marks and Simpson 1999 |
| Hybrid aspen | Salicaceae | Tree | Landscaping; wood | De Block 1990 |
| Hybrid poplar | Salicaceae | Tree | Landscaping; wood | De Block 1990 |
| Indian red wood | Meliaceae | Tree | Wood; medicinal | Chiruvella et al. 2011 |
| Lavender | Lamiaceae | Herbaceous plant | Essential oils; ornamental | Machado et al. 2014 |
| Lentil | Leguminosae* | Herbaceous plant | Seeds | Ye et al. 2002 |
| London plane tree | Platanaceae | Tree | Wood; landscaping | Alegre et al. 2015 |
| Macadamia nut | Proteaceae | Tree | Nuts | Mulwa and Bhalla 2000; Bhalla and Mulwa 2003 |
| Mango | Anacardiaceae | Tree | Fruit | Krishna et al. 2008 |
| Mexican redbud | Leguminosae* | Tree | Ornamental; landscaping | Mackay et al. 1995 |
| Neem | Meliaceae | Tree | Medicinal; vegetable | Arora et al. 2010 |
| Oak | Fagaceae | Tree | Nuts; wood | Vieitez et al. 1989 |
| Oak (white, common, northern red) | Fagaceae | Tree | Wood; ornamental | Vieitez et al. 2009 |
| Pear | Rosaceae | Tree | Fruit | Grigoriadou et al. 2000 |
| Pear | Rosaceae | Tree | Fruit | Pérez-Tornero and Burgos 2000 |
| Pear | Rosaceae | Tree | Fruit | Reed et al. 2013 |
| Pear | Rosaceae | Tree | Fruit | Jamshidi et al. 2016 |
| Pear | Rosaceae | Tree | Fruit | Wada et al. 2013, 2015 |
| Pear (incl. wild pear) | Rosaceae | Tree | Fruit | Thakur and Kanwar 2011 |
| Pistachio | Anacardiaceae | Tree | Nuts | Barghchi and Alderson 1985 |
| Pistachio | Anacardiaceae | Tree | Nuts | Abousalim and Mantell 1994 |
| Pistachio | Anacardiaceae | Tree | Nuts | Barghchi and Alderson 1996 |
| Pistachio | Anacardiaceae | Tree | Nuts | García et al. 2011 |
| Pistachio | Anacardiaceae | Tree | Nuts | Nezami et al. 2015 |
| Pistachio | Anacardiaceae | Tree | Nuts | Kermani et al. 2017 |
| Pistachio | Anacardiaceae | Tree | Nuts | Nezami-Alanagh et al. 2018 |
| Pistachio | Anacardiaceae | Tree | Nuts | Nezami-Alanagh et al. 2019 |
| Pointed gourd | Cucurbitaceae | Herbaceous plant | Vegetable | Kishore et al. 2015 |
| Poplar | Salicaceae | Tree | Landscaping; wood | Kataeva et al. 1991 |
| Potato | Solanaceae | Herbaceous plant | Tubers | Sha et al. 1985 |
| Potato | Solanaceae | Herbaceous plant | Tubers | Ozgen et al. 2011 |
| Potato | Solanaceae | Herbaceous plant | Tubers | Ahmed and Palta 2017a |
| Potato | Solanaceae | Herbaceous plant | Tubers | Ahmed and Palta 2017b |
| Quince | Rosaceae | Tree | Fruit | Singha et al. 1990 |
| Raspberry | Rosaceae | Woody shrub | Fruit | Amalia et al. 2014 |
| Rhododendron | Ericaceae | Tree | Ornamental | Compton and Preece 1988 |
| Rhododendron | Ericaceae | Woody shrub | Ornamental | Norton and Norton 1985 |
| Rice flower | Thymelaeaceae | Woody shrub | Endangered; ornamental | Offord and Tyler 2009 |
| Rose | Rosaceae | Woody shrub | Ornamental | Park et al. 2016 |
| Rose moss | Portulacaceae | Herbaceous plant | Ornamental; medicinal | Srivastava and Joshi 2013 |
| Rosewood | Leguminosae* | Tree | Wood | Lakshmi Sita and Raghava Swamy 1993 |
| Tarragona willow | Salicaceae | Woody shrub | Ornamental | Amo-Marco and Lledo 1996 |
| Tea | Theaceae | Woody shrub | Leaves | Kataeva et al. 1991 |
| Tree peony | Paeoniaceae | Tree | Ornamental | Wang and van Staden 2001 |
| White saxaul | Amaranthaceae | Woody shrub | Soil stabilization | Kurup et al. 2018 |
| Wild begonia | Begoniaceae | Woody shrub | Medicinal, ornamental | Kumari et al. 2017 |
| Wild rose | Rosaceae | Woody shrub | Ornamental | Misra and Chakrabarty 2009 |
| Wych/Scotch elm | Ulmaceae | Tree | Wood; landscaping | Mirabbasi and Hosseinpour 2014 |

* Leguminosae (alt nom. Fabaceae) Turland et al. (2018)

**Supplementary Table 2** Mineral nutrient content of the different plant tissue culture media used in STN studies

|  | | ARM | B5 | Cheng | DKW | MS | NN | QL | WPM |
| --- | --- | --- | --- | --- | --- | --- | --- | --- | --- |
| Macro nutrients  (mM) | NH_4_^+^ | 5.0 | 2.0 | 20.6 | 17.7 | 20.6 | 9.0 | 5.0 | 5.0 |
|  | K^+^ | 4.8 | 24.7 | 20.05 | 20.0 | 20.0 | 9.9 | 19.8 | 12.7 |
|  | Mg^2+^ | 1.5 | 1.0 | 1.5 | 3.0 | 1.5 | 0.75 | 1.5 | 1.5 |
|  | Ca^2+^ | 3.0 | 1.0 | 3.96 | 9.3 | 3.0 | 1.5 | 5.1 | 3.1 |
|  | Na^+^ | 3.2 | 1.1 | 0.1 | 0.3 | 0.2 | 0.2 | 0.2 | 0.2 |
|  | NO_3_^-^ | 9.7 | 24.7 | 39.4 | 34.4 | 39.4 | 18.4 | 33.0 | 9.8 |
|  | PO_4_^3-^ | 2.8 | 1.1 | 1.25 | 2.0 | 1.25 | 0.5 | 2.0 | 1.25 |
|  | SO_4_^2-^ | 1.8 | 2.1 | 1.75 | 12.3 | 1.7 | 0.95 | 1.6 | 7.5 |
|  | Cl^-^ | 6.0 | 2.0 | 3.96 | 2.0 | 6.0 | 3.0 | - | 1.4 |
| Meso and micro nutrients  (µM) | Mn^2+^ | 100.0 | 59.0 | 100.0 | 198.0 | 100.0 | 100.0 | 4.5 | 132.0 |
|  | Zn^2+^ | 30.0 | 7.0 | 36.5 | 57.0 | 30.0 | 35.0 | 30.0 | 30.0 |
|  | BO_3_^-^ | 100.0 | 49.0 | - | 78.0 | 100.0 | 162.0 | 100.0 | 100.0 |
|  | Fe^2+^ | 200.0 | 100.0 | 110.0 | 122.0 | 100.0 | 100.0 | 100.0 | 100.0 |
|  | I^-^ | 1.8 | 4.8 | 5.0 | - | 5.0 | - | 0.5 | - |
|  | MoO_2_^2-^ | 1.0 | 1.0 | 1.03 | 1.6 | 1.0 | 1.0 | 1.0 | 1.0 |
|  | Co^2+^ | 0.1 | 0.1 | 0.1 | - | 0.1 | - | 0.1 | - |
|  | Cu^2+^ | 0.1 | 0.1 | 0.1 | 1.0 | 0.1 | 0.1 | 0.1 | 1.0 |
|  | Ni^2+^ | - | - | - | 0.02 | - | - | - | - |
| Ca/K | | 0.63 | 0.04 | 0.197 | 0.47 | 0.15 | 0.15 | 0.26 | 0.24 |
| Ca/Mg | | 2.00 | 1.00 | 2.64 | 3.10 | 2.00 | 2.00 | 3.40 | 2.07 |
| NH_4_/NO_3_ | | 0.52 | 0.08 | 0.52 | 0.51 | 0.52 | 0.49 | 0.15 | 0.51 |
| NH_4_/Ca | | 1.67 | 2.00 | 5.2 | 1.90 | 6.87 | 6.00 | 0.98 | 1.61 |
| Total N (mM) | | 14.70 | 26.70 | 60.0 | 52.10 | 60.00 | 27.40 | 38.00 | 14.80 |
| Compared to MS | | | | | | | | | |
| Ca^2+^ | | 1.00 | 0.33 | 1.32 | 3.10 | 1.00 | 0.50 | 1.70 | 1.03 |
| BO_3_^-^ | | 1.00 | 0.49 | - | 0.78 | 1.00 | 1.62 | 1.00 | 1.00 |
| NH_4_^+^ | | 0.24 | 0.10 | 1.00 | 0.86 | 1.00 | 0.44 | 0.24 | 0.24 |
| NO_3_^-^ | | 0.25 | 0.63 | 1.00 | 0.87 | 1.00 | 0.47 | 0.84 | 0.25 |
| Mg^2+^ | | 1.00 | 0.67 | 1.00 | 2.00 | 1.00 | 0.50 | 1.00 | 1.00 |
| K^+^ | | 0.24 | 1.24 | 1.025 | 1.00 | 1.00 | 0.50 | 0.99 | 0.64 |

ARM, Anderson’s rhododendron medium (Anderson, 1984); B5, Gamborg et al. (1968); Cheng (1975); DKW, Driver and Kuniyuki (1984); MS, Murashige and Skoog (1962); NN, Nitsch and Nitsch (1969); QL, Quoirin and Lepoivre (1977); WPM, McCown and Sellmer (1987)
