# Supplementary material for: Shoot tip necrosis of in vitro plant cultures: a reappraisal of possible causes and solutions
Source: Planta. 2020 Sep 3;252(3):47. doi: 10.1007/s00425-020-03449-4 (PMC7471112; doi:10.1007/s00425-020-03449-4)
Supplement: Supplementary file 2 — Supplementary file2 (DOC 167 kb) [file 425_2020_3449_MOESM2_ESM.doc]

**Supplementary Figure 1:** Relative number of publications (data derived from Table 1) for different plants (based on growth habit) displaying STN


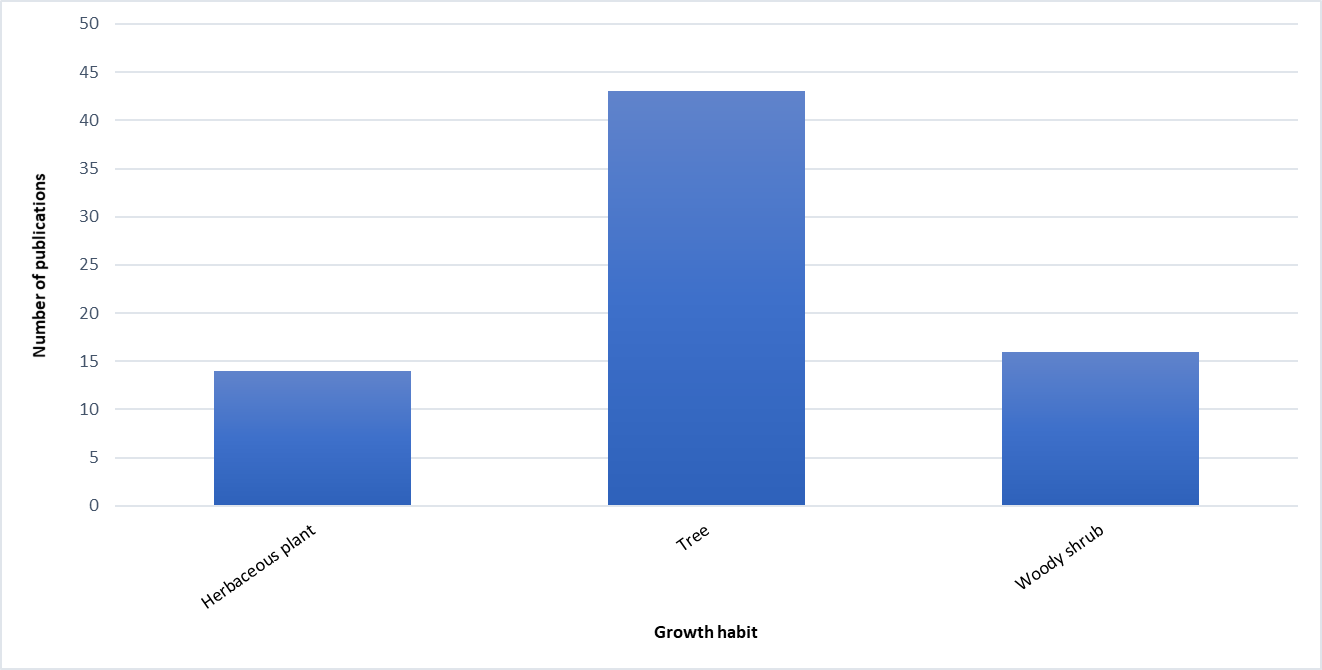


**Supplementary Figure 2:** Relative number of publications (data derived from Table 1) for different plants (based on common names) displaying STN

**
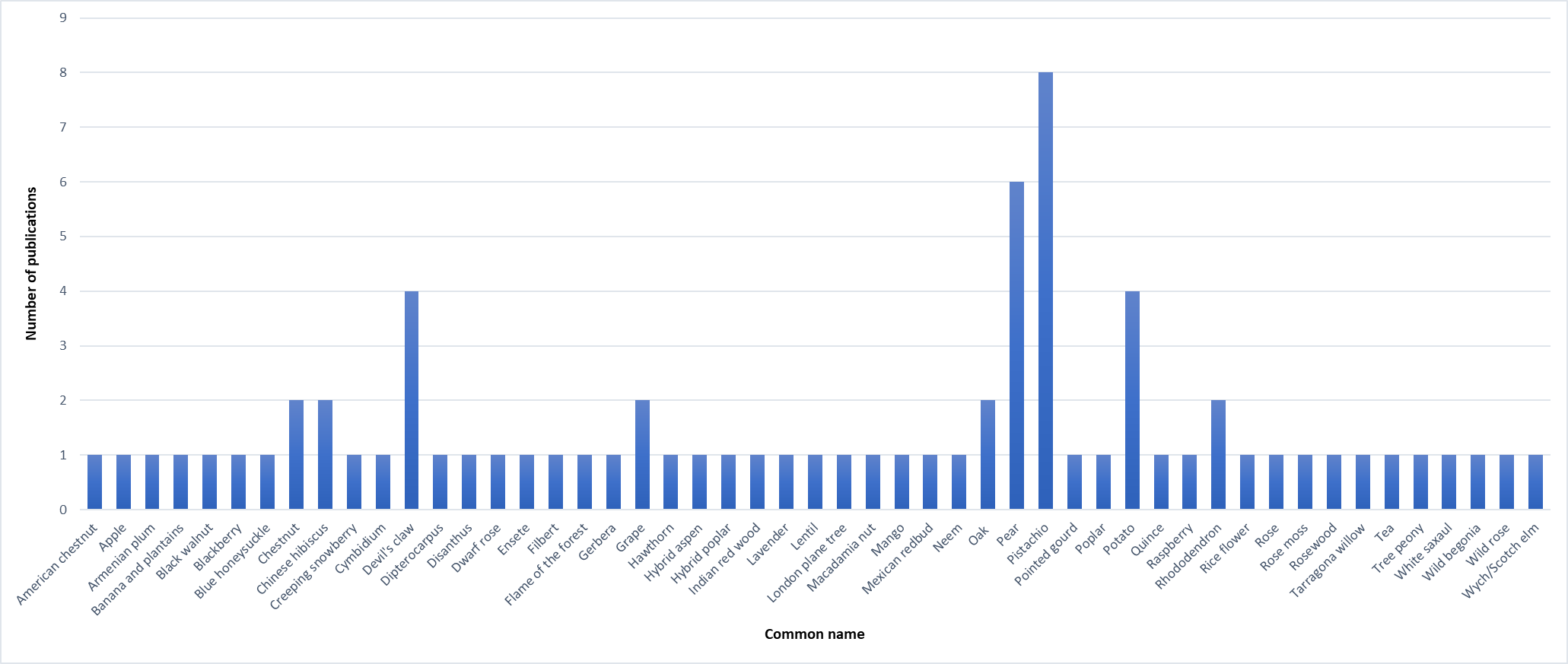
**

**Supplementary Figure 3:** Relative number of publications (data derived from Table 1) for different plants (based on plant family) displaying STN

**
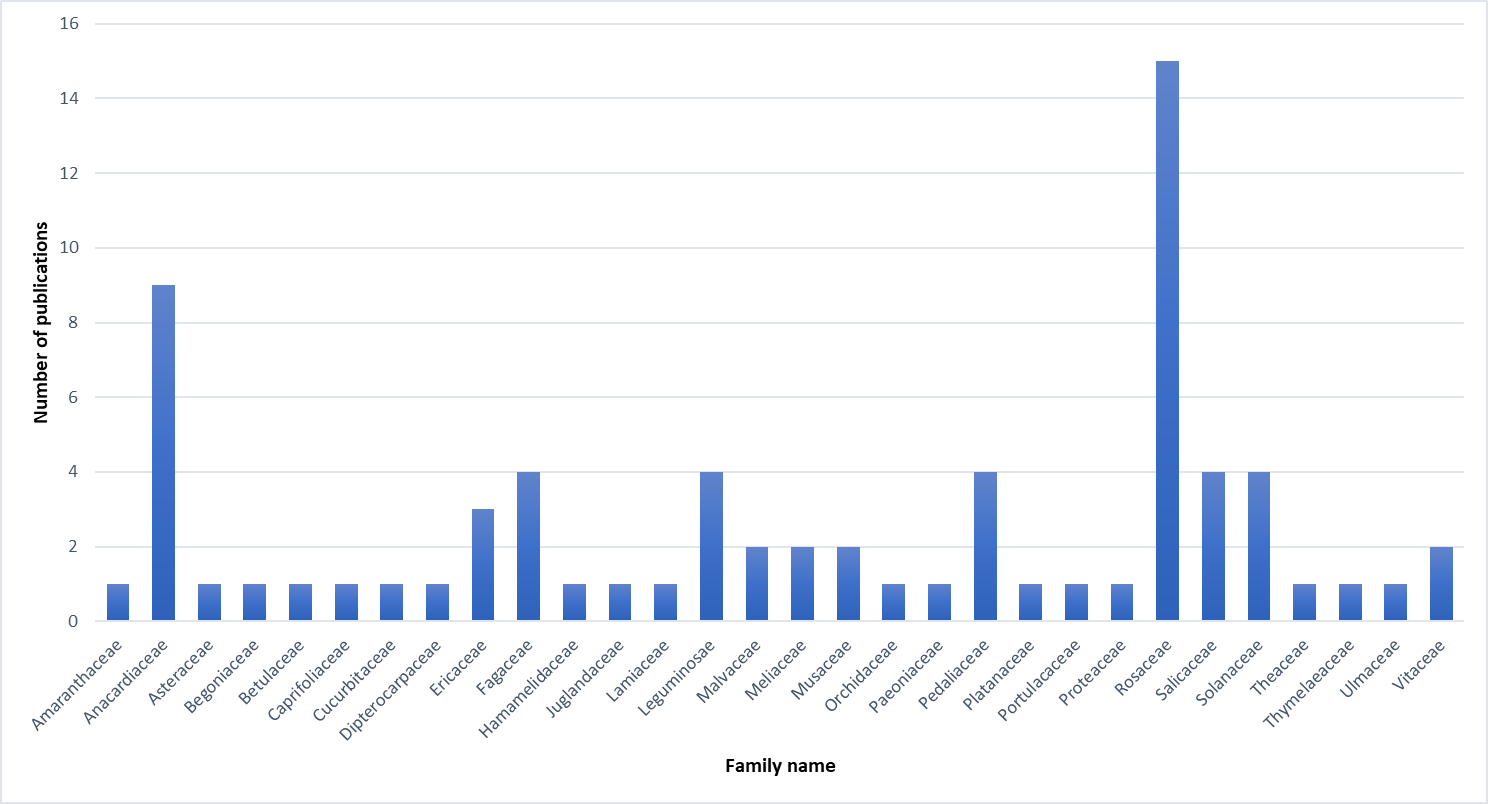
**
